# Only Children and Low Family Size Ideals: Did the One-Child Policy Create a “Low-Fertility Trap” in China?

**DOI:** 10.1007/s10680-025-09755-5

**Published:** 2025-11-14

**Authors:** Shuang Chen, Stuart Gietel-Basten

**Affiliations:** 1 Department of Social Policy, London School of Economics and Political Science, Houghton Street, London, WC2A 2AE UK; 2 Division of Social Science, The Hong Kong University of Science and Technology, Hong Kong SAR, China

**Keywords:** Low fertility, Ideal family size, Sibship size, China, One-child policy

## Abstract

The factors that shape fertility preferences—and their transition to reality—have been widely discussed. However, very few empirical studies have estimated the causal effect of sibship size on fertility preferences. Using the case of urban China, this study examines if growing up as an only child can lead to lower fertility ideals. Exploiting the introduction of the one-child policy in 1980 and using a fuzzy regression discontinuity design, the study finds that, among individuals born right around 1980, the increased probability of being an only child significantly reduces the ideal number of children and the probability of desiring two or more children. The sibship size effect not only offers a plausible explanation for low fertility ideals in urban China but also attests to a key mechanism underlying the “Low-Fertility Trap” hypothesis.

It is generally agreed that fertility preferences—whether ideals or intentions—are central to understanding fertility behaviours and outcomes. Yet, how these preferences are formed (Aassve et al., 2024; Blake, 1966; Brinton et al., 2018; Girard & Roussel, 1982; Philipov & Bernardi, 2011; Ryder & Westoff, 1971; Sobotka & Beaujouan, 2014; Trent, 1980), how they change over the life course (Heiland et al., 2008; Müller et al., 2022; Ray et al., 2018; Savelieva et al., 2021, p. 2022; Trinitapoli & Yeatman, 2018; Yeatman et al., 2013), and how they are translated into reality (Basten & Verropoulou, 2015; Bongaarts, 2001, 2002; Hagewen & Morgan, 2005; Sobotka & Beaujouan, 2014) remain to be fully understood. These questions are closely related to a central issue in low-fertility research: the discrepancy between desired and achieved family size. In most post-transitional societies, even though the total fertility rate (TFR) is well below the replacement level of 2.1, the average ideal family size^1^ remains at or above two (Bongaarts, 2001, 2002; Gietel-Basten, 2019b; Hagewen & Morgan, 2005; Sobotka & Beaujouan, 2014). This gap between ideal and actual fertility has often been taken to indicate an “unmet demand” for children (Chesnais, 1996, 2000), motivating a suite of policies designed to help individuals meet their reproductive aspirations (UNFPA, 2024).

China, however, presents a distinct case. The country recorded negative population growth for the first time in six decades in January 2023 (National Bureau of Statistics of China, 2023), and the TFR in 2022 was reported to be as low as 1.07 (Wang, 2023). There is much concern, domestically and internationally, about the demographic future of China, and much attention is currently being placed on the potential future trajectory of fertility and the possible impact of policies designed to support families. Yet unlike other post-transitional, low-fertility societies, both the TFR and average ideal family size in urban China are below two (Basten & Gu, 2013; Gietel-Basten, 2019b; Hou, 2015; Morgan et al., 2009; Zheng et al., 2018; Zhuang et al., 2020). This implies limited unmet demand and little room for policies to increase fertility (Chesnais, 1996, 2000). It also raises the concern that fertility is unlikely to recover and might decline even further (Bongaarts, 2001; Goldstein et al., 2003).

What explains the low fertility ideals in urban China? In this study, we test whether growing up as an only child can lower one’s ideal family size. Although positive *associations* between one’s sibship size (i.e., number of siblings) and fertility (Beaujouan & Solaz, 2019; Murphy, 1999, 2013; Murphy & Wang, 2001) have long been established, causal evidence remains scarce (Cools & Kaldager Hart, 2017; Kolk, 2015). Recent evidence from China shows that adult only children, on average, have lower fertility ideals and are more likely to desire only one child compared to their peers with siblings (Liu, 2025; Zhuang et al., 2020, p. 202). However, it remains unclear whether the relationship is causal. The lack of causal evidence is partly due to the empirical challenge of identifying the sibship size effect. To address this challenge, in this study, we use a fuzzy regression discontinuity (RD) design and exploit the introduction of the one-child policy (OCP) in 1980 as an exogenous source of increase in the probability of being an only child.

We find consistent evidence that the increased probability of being an only child due to the introduction of the OCP has led to a significant decrease in the ideal family size. Our findings contribute to the ongoing debates about the long-term consequences of the OCP and its implications for China’s demographic future. Our findings also highlight an intergenerational mechanism consistent with the “Low-Fertility Trap” hypothesis (Goldstein et al., 2003; Lutz et al., 2006; Testa & Grilli, 2006), contributing to a broader understanding of fertility preferences in low-fertility societies.

## Background and Context

### Sibship Size and Fertility Preferences: Theoretical Pathways

The positive correlation between family sizes in successive generations has long been observed and theorised (Bernardi, 2016; Murphy, 1999, 2013; Murphy & Wang, 2001). Sociological explanations have focused on the socialisation process in the family one grows up in. In an article dating back to 1965, Duncan et al., (1965, p. 514) postulated that children “tend to recreate a familial setting resembling the one in which they grew up in order to mobilize familiar resources, relationships, and roles.” When children form their own families as adults, they learn from social interactions in the families they grow up in and tend to “recapitulate” role relationships from this early experience (Duncan et al., 1965, p. 514). Because social interactions and role relationships depend partly on the number of siblings, there is a tendency to reproduce the size of the family one grew up in (Duncan et al., 1965, p. 515). Importantly, Duncan et al. noted that this tendency to reproduce family size “could operate independently of any direct transmission of norms about family size” (1965, p. 515). Consistent with Duncan et al.’s (1965) theory, empirical studies have found the intergenerational association of family sizes or family size preferences to be stronger among firstborns (Hendershot, 1969; Johnson & Stokes, 1976; Morosow & Kolk, 2020), those who are more satisfied with the family they grew up in (Hendershot, 1969; Johnson & Stokes, 1976), and those who experience less intergenerational changes in lifestyle or educational mobility (Johnson & Stokes, 1976).

Axinn et al. (1994) extended Duncan et al.’s theory by highlighting the role of parents’ preferences in the socialisation process. While Duncan et al. (1965) assumed that children’s fertility preferences are formed by directly learning from parents’ fertility behaviours and social interactions with siblings, Axinn et al. (1994) posited that parents’ fertility behaviours shape their own attitudes and preferences, which in turn influence children’s preferences. Empirical research has shown a direct influence of parents’ preferences, attitudes, and ideals on children’s preferences and family formation behaviours (Axinn et al., 1994; Barber, 2000; Jennings et al., 2012, p. 20; Thornton, 1980), and a general similarity in gender role, political, and religious attitudes between parents and children (Glass et al., 1986). Axinn and colleagues (Axinn & Thornton, 1996; Axinn et al., 1994) further demonstrated that parents’ attitudes and preferences mediate part of the link between their behaviour and their children’s attitudes and preferences. However, parents’ behaviour remains a strong predictor of their children’s attitudes and preferences, suggesting that social interaction and learning, as suggested by Duncan et al. (1965), may still be at play.

Similar mechanisms linking parents’ fertility behaviours and children’s fertility preferences can be inferred separately from the theory of planned behaviour (TPB) used to understand fertility preferences (Aizen & Klobas, 2013; Ajzen, 1991, 2012). According to the TPB, parents’ fertility behaviours shape our fertility preferences in two ways. First, by directly observing parents’ fertility behaviours, we perceive the positive and negative consequences of having a child, and these *behavioural beliefs* shape our attitude toward having a child. Second, we refer to parents’ fertility behaviours to perceive their expectations and preferences, and these *normative beliefs* produce a social pressure to have more or fewer children. Unlike Duncan et al.’s theory, which suggests that family size preferences “reproduce” the sibship size (1965), according to the TPB, one can have similar or opposite family size preferences from the size of the family they grew up in. Negative associations between sibship size and family size preferences have been documented in empirical studies, particularly among women (Anderton et al., 1987; Cools & Kaldager Hart, 2017). For example, Anderton et al (1987) observed that Mormon daughters with exposure to a large number of siblings have smaller family sizes, suggesting their desire to avoid rather than repeat their mother’s experiences. Fasang and Raab (2014) critically argued that intergenerational effects on family formation should not be narrowly equated with “similarity”. Their study presented compelling evidence that the intergenerational patterns comprise not only “similarity”, but also systematic deviation and contrast between parents’ and children’s family-formation trajectories, including but not limited to childbearing (Fasang & Raab, 2014).

Distinct from the socialisation process, sibship size can also influence fertility preferences through parental investment and educational attainment: smaller sibship size is often assumed to increase parental investment in children, and increased parental investment leads to increased educational attainment of children, which in turn changes their fertility preferences. Both the “resource dilution” hypothesis in sociology (Anastasi, 1956; Blake, 1981, 1989) and the “quantity-quality trade-off” in economics (Becker, 1960; Becker & Tomes, 1976) predict that smaller sibship size should lead to increased parental investment and child educational attainment. However, the empirical evidence on the relationship between sibship size and educational attainment is mixed (J. Angrist et al., 2010; Black et al., 2005; Kugler & Kumar, 2017; Li et al., 2008; Ponczek & Souza, 2012; Qian, 2009; Rosenzweig & Wolpin, 1980; Rosenzweig & Zhang, 2009). In the Chinese context, research has found positive, null, and negative effects of sibship size on educational attainment (Li et al., 2008; Qian, 2009; Rosenzweig & Zhang, 2009). Equally ambiguous is the impact of educational attainment on family size preferences in post-transitional societies. Theoretically, educational attainment can increase the opportunity cost of raising children, reducing the demand for children. But it can also increase income, allowing one to afford more children. Empirically, emerging evidence shows that the negative relationship between education and fertility has weakened or even reversed in post-transitional societies (Fort et al., 2016; Hazan & Zoabi, 2015; Hoem & Hoem, 1989; Kravdal, 1992; Kravdal & Rindfuss, 2008; Nisén et al., 2021; Testa, 2014; Wood et al., 2014, 2020). A recent systematic review of studies in China has found negative, null, and positive relationships between education and fertility (Gao & Wang, 2025). Taken together, while sibship size can influence fertility preference through parental investment and educational attainment, the direction of the influence is unclear, and the empirical evidence is mixed both in terms of the sibship effect on educational attainment and the educational effect on fertility preference.

### Endogeneity of Sibship Size

A large body of empirical literature has documented a persistent positive association between parents’ and children’s fertility across post-transition societies, even though disagreement remains about whether this intergenerational association has become stronger or weaker over time (Beaujouan & Solaz, 2019; Murphy, 1999, 2013; Murphy & Wang, 2001). There is also direct evidence of a positive association between parents’ fertility and children’s fertility preferences (Axinn et al., 1994; Hendershot, 1969; Régnier-Loilier, 2006). However, more recent studies have called into question whether the relationship between sibship size and fertility or fertility preferences can be interpreted causally. Using Swedish register data, Kolk (2015) finds that an exogenous increase in sibship size due to the birth of a twin does not lead to any changes in fertility, suggesting that the intergenerational correlation in fertility is spurious. The strongest evidence of a causal effect of sibship size on fertility comes from Norway (Cools & Kaldager Hart, 2017). Using high-quality register data and the sex composition of the first two children as an instrumental variable, Cools and Kaldager Hart (2017) find that an additional sibling has a positive effect on men’s fertility but a negative effect on women’s fertility. They speculate that women witness the family-work conflict faced by their mothers as a result of the increased family size, and consequently, limit their own number of children when they reach adulthood (Cools & Kaldager Hart, 2017).

Identifying the causal effect of sibship size on fertility preferences is challenging because a variety of mechanisms can work independently or in tandem to produce the same observed intergenerational relationship. In Duncan et al.’s own words, sibship size is “a single specific variable in the socialization environment” and “takes its place among a number of other factors” (1965, p. 515). Firstly, the relationship between sibship size and fertility preferences can be confounded by family background characteristics. Even though many studies have controlled for measures of family socioeconomic status (Beaujouan & Solaz, 2019; Murphy & Knudsen, 2002; Murphy & Wang, 2001), omitted or unobservable family background characteristics can still confound the relationship between sibship size and fertility preferences. In fact, Dahlberg (2013) used Swedish register data to show that parents’ number of children only explains a minor proportion of the sibling correlations in fertility, suggesting that the similarity in siblings’ fertility behaviours stems from family background characteristics other than parents’ fertility. Secondly, growing research has shown that parents transmit to children a genetic disposition to having children, which affects both fertility behaviours and motivations for having children (Kohler et al., 1999; Mills & Tropf, 2015; Nisén et al., 2013). This means that even with extensive control variables, correlations between sibship size and fertility preferences can still be confounded by unobservable factors and thus cannot be interpreted causally. Thirdly, parents’ own preferences, norms and attitudes can both determine their own fertility behaviours and directly influence children’s preferences, thus confounding the sibship size effect. In theory, direct norm transmission (Axinn et al., 1994; Barber, 2000; Glass et al., 1986; Jennings et al., 2012, p. 20; Thornton, 1980) is a distinct process from social interactions with siblings or reproducing parents’ behaviours (Duncan et al., 1965, p. 515). Empirically, however, it is difficult to distinguish the two processes, which can operate independently but concurrently.

Two identification strategies have been used to estimate the causal effect of sibship size on fertility: one uses the birth of twins and the other uses sex composition of the first two children (Cools & Kaldager Hart, 2017) as an exogenous increase in family size. These two strategies were originally proposed and used to study the sibship size effect on educational attainment (Angrist et al., 2010; Conley & Glauber, 2006; Rosenzweig & Wolpin, 1980). However, neither strategy allows us to study the effect of being an only child. Furthermore, prior research suggested that twin births may not be a valid instrument in the Chinese context because couples purposely misreported their nontwin children as twins in the census to avoid the punishment for violating the OCP (Huang et al., 2015). In this study, we exploit the introduction of China’s OCP in 1980 and use a fuzzy RD design to identify the causal effect of being an only child (i.e., having no siblings). A similar design has previously been used by Cameron et al. (2013), who showed that being an only child as a result of the OCP has a causal impact on behavioural outcomes such as altruism, trust, trustworthiness, attitudes toward risk, and competitiveness. In a different context, Behrman (2015) leveraged the introduction of the Universal Primary Education (UPE) policies in three African countries and found that a discontinuous jump in years of schooling led to a decrease in fertility ideals.

### The Long Shadow of the OCP

In late 1979, following the second session of the 5th National People’s Congress, several provincial governments started introducing financial, administrative, and legislative measures restricting couples to a single child (Liang, 2014). On 25 September 1980, the OCP was announced nationally in the form of an Open Letter to members of the Chinese Communist Party and the Communist Youth League (Feng et al., 2013). Prior to the introduction of the OCP, there was a nationwide anti-natalist push in the 1970s through the *wan*, *xi*, *shao* policy, which aimed to limit fertility by promoting *later* marriage*, longer* birth spacing, and *fewer* births (Banister, 1987).

Since 1984, there have been multiple reforms relaxing the OCP (Greenhalgh, 1986, 2008; Gu et al., 2007; Zeng & Hesketh, 2016) until, in 2015, it was officially ended and replaced by a national two-child policy (BBC News, 2015). In 2021, a three-child policy was announced (BBC News, 2021). At the same time, Chinese governments at central and local levels have introduced a wide range of measures aimed at stimulating fertility, from “baby bonuses” (Yeung, 2021) to “prevention of unwanted pregnancies and a decrease in non-medical abortions” (Global Times, 2022). Nevertheless, neither such incentives nor the relaxation of birth restrictions has had a significant impact on raising fertility (Attané, 2016; Basten & Jiang, 2015; Guo et al., 2019; Li et al., 2019), and fertility remains low in China. The latest *World Population Prospects* estimated that the Chinese TFR is 1.00 in 2023 (United Nations Department of Economic and Social Affairs, Population Division, 2024): one of the lowest in the world.

These recent trends have prompted renewed attention to the long-term consequences of China’s birth control policies. While early accounts often assumed the birth control policies alone to be the primary driver of almost the entire fertility landscape, more recent scholarship has challenged this narrative, reappraising the policy’s role and emphasizing the broader social, economic, and institutional changes (Basten & Jiang, 2014; Cai, 2010; Cai & Feng, 2021; Chen & Gietel-Basten, 2022; Feng et al., 2013; Goodkind, 2017; Greenhalgh, 2008, 2018; Wang et al., 2018; Zhao & Zhang, 2018). At the same time, research has increasingly looked beyond “the number of births averted” and examined the wider, far-reaching social consequences of the OCP (Cai & Feng, 2021; Zeng & Hesketh, 2016).

One important line of inquiry concerns the impact of the OCP on shaping fertility preferences (Basten & Gu, 2013; Chen & Gietel-Basten, 2022; Merli & Morgan, 2011; Merli & Smith, 2002; Nie & Wyman, 2005; Whyte & Gu, 1987; Zhang, 2007; Zhenzhen et al., 2009). Particularly, the limited effectiveness of recent policy reforms at raising fertility has led some scholars to question whether China’s population policies have created enduring norms of small family sizes (Basten & Jiang, 2015). A meta-analysis (Hou, 2015) estimates the mean ideal family size in the 2000s to be 1.67, 1.50 in urban areas and 1.82 in rural areas. The average individual ideal family size remains low even after taking into account the effect of policy restrictions on survey responses (Chen & Gietel-Basten, 2022). Building on this body of work, our current study examines whether growing up as an only child can lower one’s ideal family size. If so, this would suggest that population policies may have an intergenerational impact on fertility preferences, and that sibship size could serve as one explanation for why the average ideal family size in China is below the two-child norm characterising other post-transitional societies.

## Data and Method

### Fuzzy RD Design

The basic idea behind applying the fuzzy RD design is that the introduction of the OCP created a discontinuous “jump” in the probability of being an only child at the cutoff birth year of 1980. If we also observe a discontinuous “jump” in fertility ideals at the cutoff birth year of 1980, and if there appears to be no other reason for fertility ideals to be a discontinuous function of one’s birth year, we can attribute the “jump” in fertility ideals to the “jump” in probability of being an only child induced by the introduction of the OCP. For the RD design to be valid, all other factors determining fertility ideals must evolve “smoothly” with respect to the birth year (Imbens & Lemieux, 2008, p. 616).

One compelling feature of the RD design is that it allows other factors to be associated with both birth year and fertility ideals as long as the association does not “jump” at the same cutoff year of 1980 as the OCP. In fact, the RD design allows birth year itself to be associated with fertility ideals as long as the association is smooth (Imbens & Lemieux, 2008). This assumption is much weaker than what is required by many other causal inference methods, making the RD design a suitable approach to studying the effect of sibship size change amidst the many institutional, socioeconomic, and ideational changes in the Chinese context.

The RD design can also be formulated as a local randomised experiment at the cutoff (Lee, 2008; Lee & Lemieux, 2010, p. 289): if individuals have imprecise control over birth year, the OCP is “as good as” randomly assigned around the cutoff. The local random assignment allows us to identify the effect of the OCP at the cutoff birth year of 1980. Formulating the RD design as a local randomised experiment means we can empirically test and verify its validity, including whether pre-treatment characteristics are continuous in birth year at the cutoff (Lee, 2008; Lee & Lemieux, 2010, p. 296), akin to checking whether the treatment and control groups are balanced in a randomized control trial. The ability to empirically test its internal validity is another compelling feature of the RD design, distinct from other causal inference methods (Lee & Lemieux, 2010, p. 306).

In our study, we use a *fuzzy* RD design because one-child families existed even before 1980, and not everyone born in or after 1980 is an only child. While the OCP was introduced nationally in 1980, it was met with resistance and never perfectly enforced (Greenhalgh, 1986). As such, we can only state that the introduction of the OCP increased the *probability* of being an only child. If we see the fuzzy RD design as a local randomised experiment, all individuals born in or after 1980 are *assigned* the treatment, but the compliance is imperfect, and not all of them *receive* the treatment. The fuzzy RD estimate is calculated by first estimating the effects of being *assigned* the treatment on the outcome and on the probability of receiving the treatment, respectively, and then taking the ratio of the two. In this case, we estimate the effects of OCP assignment on fertility ideals and on the probability of being an only child, respectively, and then take the ratio of the two to obtain the estimated effect of being an only child on fertility ideals. This is analogous to an instrumental variable estimator (Hahn et al., 2001), which is commonly used to analyse random experiments with imperfect compliance.

### Model Specifications

Formally, let ${T}_{j}=1[{x}_{j}\ge 1980]$ denote the OCP assignment status, where ${x}_{j}$ indicates the birth year (i.e., the running variable). Because birth year is discrete, ${x}_{j}$ can take on $J$ unique values $({x}_{1},\dots , {x}_{J})$. Let ${D}_{ij}$ denote whether individual $i$ born in year ${x}_{j}$ *receives* the treatment: ${D}_{ij}$ equals 1 if the individual is an only child, and zero otherwise. We specify a first-stage equation as follows, estimating the effect of OCP assignment on the probability of being an only child:

1 $${D}_{ij}={\beta }_{1}{T}_{j}+h\left({x}_{j}\right)+{\varepsilon }_{ij},$$

where $h(\cdot )$ is a continuous function describing the relationship between year of birth and probability of being an only child. Our parameter of interest is ${\beta }_{1}$, which estimates the discontinuous jump in the probability of being an only child at the cutoff. We then specify a reduced form equation estimating the effect of OCP assignment on the outcome:

2 $${Y}_{ij}={\beta }_{2}{T}_{j}+g\left({x}_{j}\right)+{\eta }_{ij},$$

where ${Y}_{ij}$ is the fertility ideal of individual $i$ born in year ${x}_{j}$, and $g(\cdot )$ is a continuous function describing the relationship between year of birth and fertility preferences. The parameter of interest, ${\beta }_{2}$, estimates the discontinuous jump in fertility preferences at the cutoff. The fuzzy RD estimate is the ratio of the reduced-form coefficient to the first-stage coefficient:

3 $$\tau =\frac{{\beta }_{2}}{{\beta }_{1}}.$$

It identifies the average effect of being an only child due to the introduction of the OCP for individuals born in or near 1980.

The validity of the RD estimate relies crucially on the functional form assumed for $h(\cdot )$ and $g(\cdot )$, especially when the running variable is discrete. This is because, to estimate the local treatment effect at the cutoff, the RD design draws on data further away from the cutoff and uses regression to estimate the conditional expectation of the outcome variable at the cutoff by extrapolation (Lee & Lemieux, 2010, p. 285). Furthermore, when the running variable is discrete, bandwidth selection methods such as the mean squared error-optimal estimator (Calonico et al., 2020) are not appropriate, and parametric extrapolation is unavoidable (Cattaneo et al., 2023, p. 63). Following the recommendations of Lee and Lemieux (2010, p. 284), in this study, we use a range of specifications, including higher-degree polynomials fit to data points far away from the cutoff and local linear regressions fit to data points close to the cutoff, to show that our results are stable across the different functional forms and analytic windows.

### Data

We use data from the Chinese General Social Survey (CGSS), a nationally representative, repeated cross-sectional survey (Bian & Li, 2012). The fuzzy RD estimator as described above requires that we have data on birth year, sibship size, and fertility preferences. However, only two waves of the CGSS contain all three variables, which leaves us with too small a sample to generate reliable estimates. To circumvent this problem, we use the two-sample instrumental variables (TSIV) procedure (J. D. Angrist & Krueger, 1992; Inoue & Solon, 2010), which requires only one data set containing birth year and sibship size, and another dataset containing birth year and fertility preferences for the same cohorts.

To estimate the first-stage equation (Eq. 1), we pool data from CGSS 2006, 2008, and 2017 (Family Module), which collected information on how many siblings the respondent ever had. We removed 26 individuals (out of 20,283, 0.01%) with missing values on sibship size. To estimate the reduced form (Eq. 2), we pool data from CGSS 2006 (Family Module), 2010, 2013, 2015, 2017, 2018 and 2021, which contained information about individual ideal family size. Specifically, the survey asked the question: “In the absence of policy restrictions, how many children do you wish to have?” Fertility ideals measured by this question have been shown to predict actual reproductive behaviours in China even in the presence of family size restrictions (Jiang et al., 2016; Merli & Smith, 2002). We top-coded the ideal family size at 5 children (to keep consistent with the CGSS 2006 response categories) and removed 2,422 individuals (out of 70,913, 3%) with missing values on fertility ideals.

For both datasets, we restrict the analytic sample to Han adults aged below 50 at the time of the survey with non-agricultural *hukou* (i.e., household registration), for whom the OCP was more strictly enforced (Cai, 2010; Zhang, 2017, p. 426). Our main analyses focus on two windows: individuals born between 1969 and 1990, and individuals born between 1975 and 1984. The dataset with sibship size measure (i.e., the “first-stage” dataset) consists of 3,450 individuals born between 1969 and 1990 and 1,577 individuals born between 1975 and 1984. The dataset with the personal ideal family size measure (i.e., the “reduced form” dataset) consists of 8,696 individuals born between 1969 and 1990 and 3,952 individuals born between 1975 and 1984. Table 1 describes the analytic samples used in the main analyses. More birth-year-specific descriptive statistics are presented and discussed in detail in the next section. The exact sample size and characteristics also vary depending on the analytical choices, as detailed below.

**Table 1:** Descriptive statistics of the two datasets used for estimating the first-stage and reduced-form equations, respectively

	Born 1969 to 1990	Born 1975 to 1984
*“First-stage” dataset*		
Number of siblings	1.42	1.17
	(1.45)	(1.25)
Number of siblings (%)		
0 (i.e., only child)	32	36
1	28	32
2	21	20
3 or more	19	12
Age	29.97	27.72
	(6.87)	(4.48)
Mother has high school degree or above (%)	14	14
Mother is a member of the communist party (%)	3	3
Non-agricultural hukou at birth (%)	72	70
Survey year (%)		
2006	50	51
2008	36	35
2017	14	14
Number of individuals	3,450	1,577
**“***Reduced form” dataset*		
Personal ideal family size	1.68	1.68
	(0.69)	(0.68)
Personal ideal family size (%)		
0	4	3
1	32	32
2	60	60
3 or more	5	5
Age	35.5	34.9
	(7.00)	(4.88)
Mother has high school degree or above (%)	23	22
Mother is a member of the communist party (%)	6	7
Non-agricultural hukou at birth (%)	66	66
Survey year (%)		
2006	7	8
2010	19	20
2013	18	19
2015	14	13
2017	17	16
2018	17	17
2021	7	8
Number of individuals	8,696	3,952

Standard deviation in parenthesis. “First-stage” dataset is pooled from CGSS 2006, 2008, and 2017 (Family Module). “Reduced form” dataset is pooled from CGSS 2006 (Family Module), 2010, 2013, 2015, 2017, 2018 and 2021

## Validity of the Fuzzy RD Design

### First-Stage: Discontinuity in Probability of Being an Only Child

**Fig. 1:**
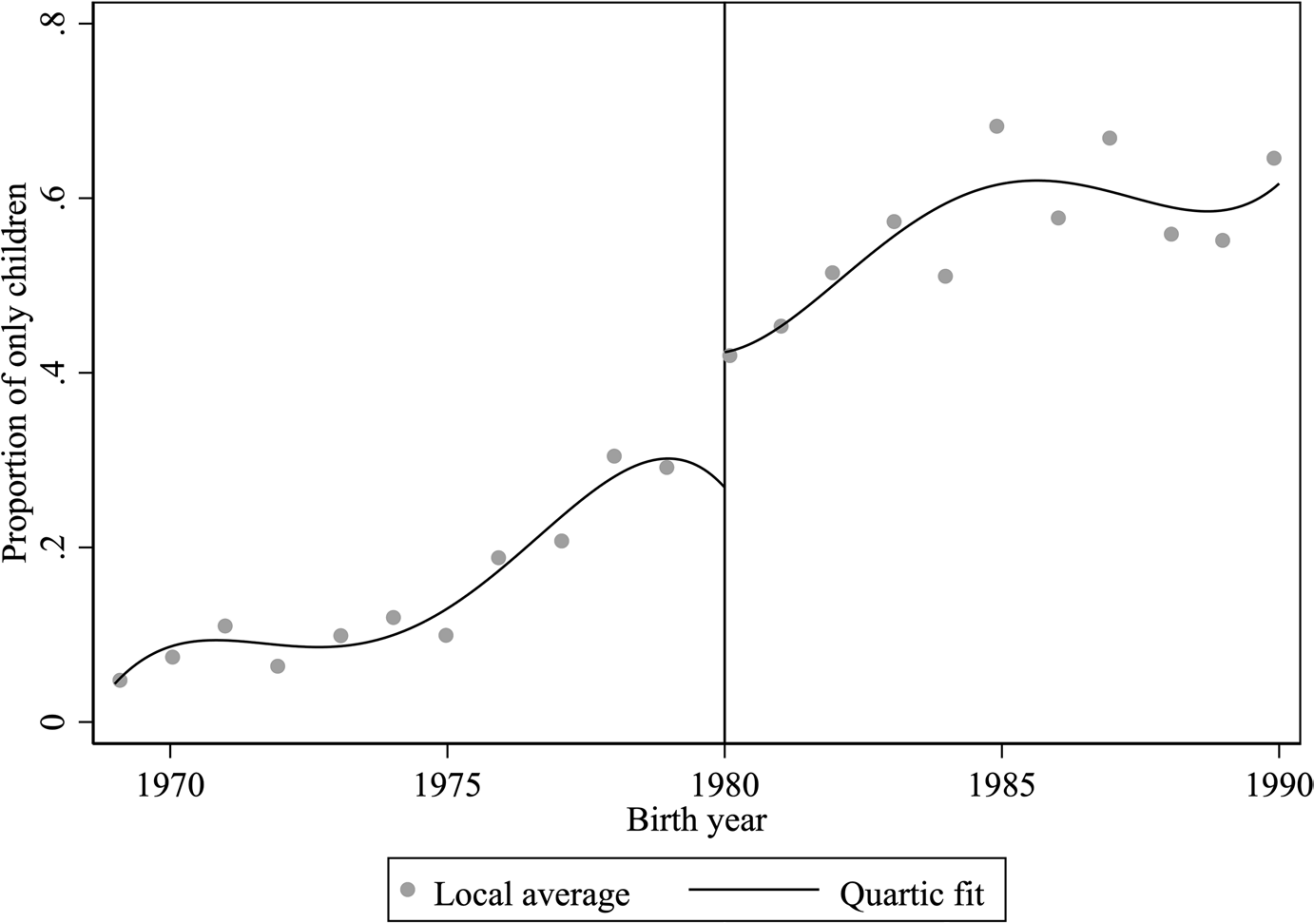
Probability of being an only child by birth year, local average and quartic polynomial fit. Note: The dots are local averages, and the curved line plots the fitted values from a quartic regression model estimated separately on each side of the cutoff. The OCP took effect in 1980, indicated by the vertical line. Source: CGSS 2006, 2008, and 2017 (Family Module)

For our analytic strategy to work, we need to first show that there is indeed a “jump”, or discontinuity, in the probability of being an only child when the OCP took effect in 1980. Figure 1 plots the share of only children by birth year between 1969 and 1990. The curved line plots the fitted values from a quartic regression model estimated separately on each side of the cutoff. According to Fig. 1, while the proportion of only children had been on the rise even before 1980 (due to the anti-natalist policies already in place in the early 1970s), there is a clear jump in the probability of being an only child when the OCP took effect in 1980. The quartic polynomials fit to each side of the cutoff, as illustrated in Fig. 1, predict a jump of 15.5 percentage points in the probability of being an only child, from 27% right before 1980 to 42% right after. The estimates are presented in Model 1 Table 2. The discontinuity is statistically significant from zero with an F-statistic of 13.89.

**Table 2:** First-stage: Estimated discontinuity in probabilities of being an only child

	(1)	(2)	(3)
	Discontinuity in being an only child		
$\widehat{{\beta }_{1}}$	0.155**	0.117**	0.099**
	(0.042)	(0.035)	(0.028)
Birth year polynomial	Quartic	Quadratic	Quadratic
Window	[1969, 1990]	[1975, 1984]	[1975, 1984]
Covariates	No	No	Yes
F-statistic	13.89	11.41	11.30
N	3,450	1,577	1,577

Standard errors are in parentheses and clustered by birth year. All models control for birth year polynomial that is allowed to be different before and after 1980. Covariates included in Model 3 are whether mother has high school education or above, whether mother is a member of the Chinese Communist Party, whether the individual has non-agricultural hukou at birth, age of the individual at the time of the survey, fine rates on excess births in 1979 (Ebenstein, 2010), and region fixed effects. **p < 0.01

Recent literature argued against using global higher-degree polynomials for RD because they can produce unreliable point estimates at the cutoff, give more weights to data far away from the cutoff, and produce misleading confidence intervals, and recommended using local linear or quadratic models instead (Cattaneo et al., 2019; Gelman & Imbens, 2019). Therefore, in Model 2 Table 2, we limit our analysis to data within five years of the cutoff (i.e., 1975 to 1984) and fit a local quadratic regression weighted by a triangular kernel function (Cattaneo et al., 2019, p. 43) to each side of the cutoff. The triangular kernel function assigns weight to each observation so that observations closer to the cutoff are weighted more than those further away (Cattaneo et al., 2019, p. 43). The local quadratic regression model estimated a jump of 11.7 percentage points in the probability of being an only child (Model 2 Table 2). The discontinuity is statistically significant from zero with an F-statistic of 11.41.

Lee and Lemieux (2010) argued that local quadratic or linear regressions alone do not solve the functional form issues and that we should not rely on a particular specification. Following their recommendation (Lee & Lemieux, 2010, p. 284), later in the paper, we explore the sensitivity of the results to a range of windows and orders to the polynomial and show that, across model specifications, there is a statistically significant discontinuity in the probability of being an only child.

### Density of the Running Variable

One of the most important assumptions of the RD design is that individuals are unable to precisely control the running variable (Lee & Lemieux, 2010, p. 293). Suppose parents were aware that from 1980 onwards they wouldn’t be allowed to have more than one child, they may have attempted to conceive and give birth just before the policy was announced. If parents were able to precisely manipulate when their child was born, the RD design is not valid. To check if there was manipulation, we first examine a histogram of the running variable (Appendix Figure 3a), and there is no obvious sign of any sudden change in density near 1980. Next, we conduct a manipulation test (Cattaneo et al., 2020; McCrary, 2008), which treats the density as a dependent variable in a local polynomial regression and formally tests if there is any abrupt change in the density of the running variable at the cutoff. The test shows no significant change in the density at the cutoff (p-value = 0.39), and the local polynomial density estimates are plotted in Appendix Figure 3b.

### Continuity in Pre-treatment Covariates

If individuals are unable to precisely manipulate the running variable, no covariates that are determined prior to the introduction of the OCP should “jump” at the cutoff. In Appendix Figure 4, we plot five covariates by birth year: age at the time of the survey, whether the respondent’s mother had a high school degree, whether the respondent’s mother is a member of the Chinese Communist Party, whether the respondent had a non-agricultural *hukou* at birth, and the provincial variation in OCP enforcement as measured by fine rates on excess fertility in 1979. The fine rates were calculated and provided by Ebenstein (2010). We also report the estimated discontinuity from fitting quartic polynomials to each side of the cutoff as before but using each of the covariates. There is no obvious discontinuity in these pre-treatment covariates at the cutoff, and none of the estimated discontinuities is significantly different from zero, reassuring us that all the observable covariates are continuous at the cutoff.

**Fig. 2:**
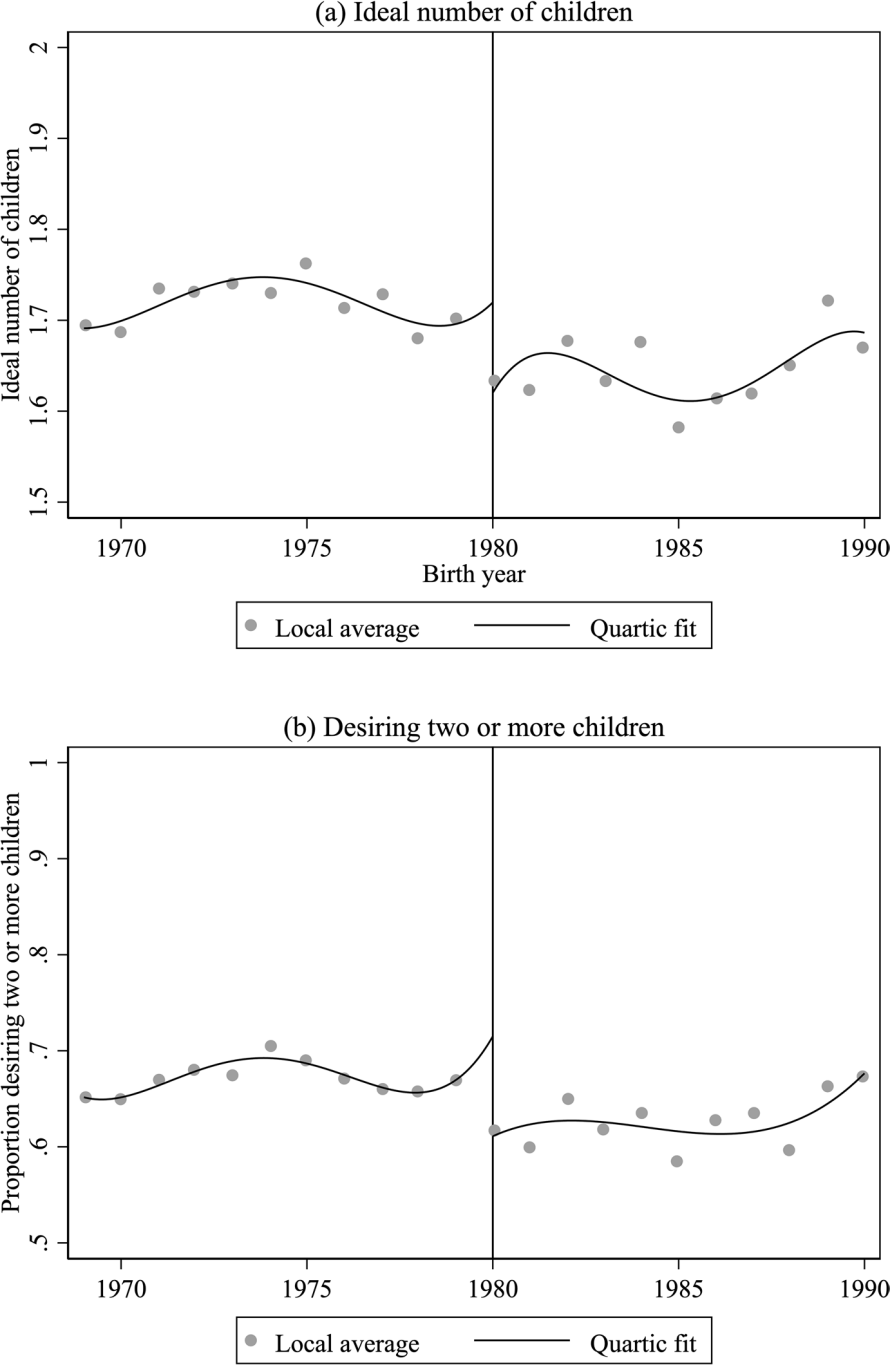
Personal ideal family size by birth year, local average and quartic polynomial fit. Note: The dots are local averages and the curved line plots the fitted values from a quartic regression model estimated separately on each side of the cutoff. The OCP took effect in 1980, indicated by the vertical line. Source: CGSS 2006 (Family Module), 2010, 2013, 2015, 2017, 2018 and 2021

Moreover, if the no-manipulation assumption holds, including covariates in our regression models should not substantially change the estimated discontinuity. Model 3 Table 2 fits a local quadratic regression weighted by a triangular kernel function to each side of the cutoff, as before, and includes all the pre-treatment covariates examined in Appendix Figure 4. Additionally, Model 3 controls for the fixed effects of six regions of China (North, Northeast, East, South-central, Southwest, and Northwest) to account for any unobservable region-level heterogeneity. Adding pre-treatment covariates and controlling for region fixed effects also help eliminate some bias resulting from using observations far away from the cutoff or inappropriate functional form and improves the precision of the estimates (Imbens & Lemieux, 2008, p. 625). As shown in Model 3 (Table 2), the estimated discontinuity in the probability of being an only child is 9.9 percentage points with covariates, a slight decrease from Model 2 without covariates, and the standard error is smaller.

## Results

### Reduced-Form: Discontinuity in Ideal Family Size

Our first-stage estimates have shown that the introduction of the OCP did indeed lead to a discontinuity in the probability of being an only child. If being an only child impacts one’s ideal family size, we expect to also see a “jump”, or discontinuous change in ideal family size when the OCP took effect in 1980. Figure 2a and b plot the personal ideal family size by birth year between 1969 and 1990, and the fitted curve is from a quartic regression model estimated separately on each side of the cutoff. Figure 2a shows a discontinuous drop in the personal ideal number of children when the OCP took effect in 1980, and the size of the drop is estimated to be 0.10 children, from 1.72 right before 1980 to 1.62 right after 1980. Similarly, Fig. 2b shows a discontinuous drop in the probability of desiring two or more children, and the size of the drop is estimated to be 10.4 percentage points, from 72% right before 1980 to 61% right after. The estimated discontinuity in personal ideal family size using quartic regression models is presented in Column 1 Table 3. These reduced-form coefficients estimate the “intent-to-treat” effect, that is, the effect of introducing the OCP in 1980 on the personal ideal family size.

**Table 3:** Reduced form: Estimated discontinuity in personal ideal family size (also known as intent-to-treat effect of the OCP on personal ideal family size)

	(1)	(2)	(3)
	Discontinuity in ideal family size		
$\widehat{{\beta }_{2}}$	− 0.099**	− 0.080**	− 0.058*
	(0.035)	(0.024)	(0.025)
	Discontinuity in desiring two or more children		
$\widehat{{\beta }_{2}}$	− 0.104***	− 0.077***	− 0.060***
	(0.016)	(0.007)	(0.006)
Birth year polynomial	Quartic	Quadratic	Quadratic
Window	[1969, 1990]	[1975, 1984]	[1975, 1984]
Covariates	No	No	Yes
N	8,696	3,952	3,952

Standard errors are in parentheses and clustered by birth year. All models control for birth year polynomial that is allowed to be different before and after 1980. Covariates included in Model 3 are whether mother has high school education or above, whether mother is a member of the Chinese Communist Party, whether the individual has non-agricultural hukou at birth, age of the individual at the time of the survey, fine rates on excess births in 1979 (Ebenstein, 2010), and region fixed effects. ***p < 0.001 **p < 0.01 *p < 0.05

In Model 2 Table 3, instead of a quartic regression model, we fit a local quadratic regression model to data points within five years of the cutoff. The ideal family size is estimated to have decreased by 0.08 on average, and the probability of desiring two or more children by 8 percentage points (Column 2 Table 2). Model 3 controls for pre-treatment covariates and region fixed effects in the local quadratic regression models, and the estimates decreased slightly in magnitude: an estimated drop of 0.06 children in personal ideal family size, and 6 percentage points in the probability of desiring two or more children (Column 3 Table 3).

### Fuzzy RD Estimates

First-stage estimates indicate that the OCP leads to a discontinuous jump in the probability of being an only child (Table 2). The reduced-form estimates indicate that the OCP also leads to a discontinuous drop in personal ideal family size, measured either by the mean ideal number of children or the probability of stating an ideal number of children of two or more children (Table 3). The fuzzy RD estimate of the effect of being an only child on ideal number of children is equivalent to taking the ratio of the reduced-form and first-stage coefficients. Table 4 presents the fuzzy RD estimates. The estimate identifies the causal effect of being an only child due to the introduction of the OCP on ideal family size for individuals born right around 1980.

**Table 4:** Fuzzy RD estimates: Estimated effect of being an only child due to the OCP on ideal family size and probability of desiring two or more children

	(1)	(2)	(3)
	Effect of being an only child on ideal family size		
$\widehat{\tau }$	− 0.641**	− 0.685***	− 0.585***
	(0.166)	(0.099)	(0.107)
	Effect of being an only child on desiring two or more children		
$\widehat{\tau }$	− 0.671**	− 0.659*	− 0.607**
	(0.210)	(0.206)	(0.153)
Birth year polynomial	Quartic	Quadratic	Quadratic
Window	[1969, 1990]	[1975, 1984]	[1975, 1984]
Covariates	No	No	Yes

Fuzzy RD estimates are calculated by taking the ratio of reduced-form and first-stage estimates, and standard errors are calculated using delta method. All models control for birth year polynomial that is allowed to be different before and after 1980. Covariates included in Model 3 are whether mother has high school education or above, whether mother is a member of the Chinese Communist Party, whether the individual has non-agricultural hukou at birth, age of the individual at the time of the survey, fine rates on excess births in 1979 (Ebenstein, 2010), and region fixed effects. ***p < 0.001 **p < 0.01 *p < 0.05

According to Model 1 fitting quartic models to data from 1969 to 1990, for individuals born right around 1980, being an only child reduces the ideal number of children by 0.64 on average and reduces the probability of desiring two or more children by 67 percentage points (Column 1 Table 4). The quartic polynomial regression models fit to data from 1975 to 1984 generate very similar results: Being an only child reduces the ideal number of children by 0.69 without covariates (Column 2 Table 4) and 0.59 with covariates (Column 3 Table 4). It reduces the probability of desiring two or more children by 66 percentage points without covariates and 61 percentage points with covariates.

## Sensitivity Analyses

### Alternative Specifications

As previously mentioned, the validity of the RD estimates relies crucially on the functional forms assumed. In the main analysis presented so far, we have fitted quartic models to individuals born between 1969 and 1990, as well as local quadratic models to those born between 1975 and 1984. Here, we check the robustness of our results by using alternative windows and polynomials, and the estimates are presented in Appendix Table 5. Columns 1 and 2 replicate the main results from Models 1 and 2 in Tables 2 and 4. Model 3 to Model 7 fit quartic models to those born 11, 10, 9, 8, and 7 years within the cutoff, respectively. Model 8 fits cubic models to those born 6 years within the cutoff. Model 9 fits local quadratic models to those born 5 years within the cutoff but with a uniform kernel. Model 10 fits local quardratic models with a triangular kernel to those born 4 years within the cutoff. Model 11 fits local linear models with a triangular kernel to those born three years within the cutoff. Excluding Models 5 and 11 (where the first stage is weak), the estimated effect of being an only child on the ideal number of children ranges from − 0.70 to − 0.55, and the estimated effect on the probability of desiring two or more children ranges from − 67.1 to − 19.3 percentage points. Across all 11 specifications, there is a negative and significant effect of being an only child on ideal family size and on the probability of desiring two or more children.

So far, we have used respondents aged below 50 in all our analyses. The ideal family size might no longer be relevant for the fertility outcomes of individuals in their late reproductive years. For those individuals, the ideal family size might also have been under- or over-reported due to post-hoc rationalisation. As a robustness check, in Model 2 Appendix Table 6, we restricted the sample to individuals aged below 40. Compared to using individuals below 50 (Model 1 Appendix Table 6), the estimated effects are very similar among individuals aged below 40: being an only child reduces the ideal family size by 0.62 children and the probability of desiring two or more children by 61 percentage points. The subsample analysis reassures us that our results hold for individuals still in their early and middle reproductive years, for whom ideal family size is more relevant and post-hoc rationalisation is less likely.

In the main analyses, we have controlled for age effects. There is concern about whether the period effect might have confounded our estimates. Particularly, before a universal two-child policy took effect in January 2016, a “partial” two-child policy was implemented first in selected cities and later nationally, allowing couples in which at least one of the partners was an only child to have two children (Zeng & Hesketh, 2016). Because one’s own only-child status directly determined eligibility to have two children during this period, its effect on fertility ideals might be driven by the period differences in policy and eligibility rather than the sibship size change across birth cohorts. In Model 3 Appendix Table 6, we control for whether the survey year was before 2016 in addition to the covariates in Appendix Fig. 4 and region fixed effects, and there is very little change to the estimates compared to not controlling for the policy period (Model 1 Appendix Table 6).

### Inference

We have clustered our standard errors at the birth year level, because it is the level at which the treatment is assigned. With a small number of clusters, however, the standard error estimates may be unreliable (Cameron & Miller, 2015). As a robustness test, we calculate alternative standard errors by collapsing the individual-level data to the level of birth year and estimating the regression models on group means weighted by the group size. The grouped estimation generates the same point estimates as the individual-level regressions but more reliable standard errors (Angrist & Pischke, 2009, p. 216). Furthermore, we base inference on a *t*-distribution with degrees of freedom equal to the number of clusters minus the number of group-constant variables, i.e., $t(G-L)$ (Cameron & Miller, 2015; Donald & Lang, 2007). This yields more conservative inference than using clustered standard errors and *t*-distribution with degrees of freedom equal to the number of clusters minus one, i.e., $t(G-1)$, which is what we have been using in the main analyses so far. Appendix Table 5 reports the t-statistics from the grouped estimation along with the more conservative p-values using $t(G-L)$. For the first stage estimates, the t-statistics are almost identical between individual-level estimation with clustered standard error and grouped estimation with robust standard errors. For the fuzzy RD estimates of the effect on ideal family size, using grouped estimation leads to larger standard errors. Using $t(G-L)$ for more conservative inference, in three out of the 11 specifications, the estimated effect of being an only child on ideal family size is no longer statistically significant. However, for the estimated effect on the probability of desiring two or more children, using grouped estimation leads to smaller standard errors, and all estimates across the 11 specifications remain statistically significant.

### Falsification Tests

Finally, our fuzzy RD estimates rely on the assumption that the introduction of the OCP affects fertility ideals through increasing the probability of being an only child. Our fuzzy RD estimates might be biased if, for those born right around 1980, the OCP affects their fertility ideals directly or through mechanisms other than the change in sibship size. Here, we present falsification tests to rule out this concern. First, the one-child policy might have affected fertility ideals by changing the sex ratio (Ebenstein, 2010; Li et al., 2011) at birth rather than sibship size. As a falsification test, we examine the sex-specific effect of being an only child on personal fertility ideals (Appendix Table 7). If our results had been driven by the change in sex ratio rather than the change in sibship size due to the OCP, we would not have observed any effect within each sex. However, Appendix Table 7 shows that there is a negative and significant jump around 1980 in men’s and women’s ideal family sizes, respectively. Moreover, being an only child due to the introduction of the OCP significantly reduces both men’s and women’s ideal family sizes. The significant sex-specific estimates reassure us that our results were not driven by the changing sex ratio at birth.

A second concern is that individuals born right before and after 1980 might be exposed to other policy changes that also affect their fertility ideals. One such reform is the higher education expansion in 1999, which might have impacted the educational attainment and, in turn, the fertility ideals of individuals born right around 1980 (Chen, 2022). To check if our results are biased by the potential impact of the higher education expansion on fertility ideals, we restrict our sample to individuals who have left school *before* 1999 and thus were not exposed to the higher education expansion. Among these individuals, we still observe a significant discontinuous decrease in fertility ideals around 1980 (Model 1 Appendix Table 8), and this decrease cannot have been driven by the higher education expansion. Similarly, in Model 2 Appendix Table 8, we examine individuals who had not attained a tertiary degree. Among these individuals who likely have not benefited from the higher education expansion, we still observe a significant discontinuous decrease in fertility ideals around 1980. These falsification tests reassure us that our results are not driven by the potential impact of the higher education expansion since the discontinuity in fertility ideals around 1980 holds even among subpopulations who were not exposed to the higher education expansion.

While we have ruled out the potential impact of changing sex ratios and higher education expansion, it is still likely that the discontinuity we observe in fertility ideals around 1980 is not due to the increased probability of being an only child but due to the direct impact of the OCP or other unobservable mechanisms. To rule out this further concern, we conduct a placebo test: among individuals who have siblings, there is no change in the probability of being an only child, and therefore, we should not observe any discontinuous drop in fertility ideals around 1980 unless other mechanisms exist. When we restrict our analyses to individuals who are not only children in Model 3 Appendix Table 8, there is indeed no significant discontinuity in either the ideal family size or the probability of desiring two or more children. In fact, the estimated coefficients are positive, suggesting that even if there were other mechanisms, they would have led to an underestimation of the negative effects of being an only child on fertility ideals. Taken together, the falsification tests in this section reassure us that the negative effects we found on fertility ideals are indeed driven by the increased probability of being an only child due to the introduction of the OCP.

## Discussion and Conclusions

In the context of China’s persistent low fertility and the government’s latest policies to move towards what it calls a “sustainable fertility rate” (People’s Congress, 2021), we sought to understand why the average ideal family size in China is below the two-child norm characterising most other post-transitional societies. Building on theories of intergenerational transmission of fertility (Bernardi, 2016; Murphy, 1999, 2013; Murphy & Wang, 2001), we test whether growing up as an only child can lower one’s fertility ideals. Exploiting the introduction of the OCP in 1980, our fuzzy RD estimates show that the increased probability of being an only child significantly reduces the ideal number of children and the probability of desiring two or more children among individuals born right around 1980. The sibship size effect thus provides a plausible explanation for why the family size ideals are low in urban China.

The sibship size effect on fertility ideals revealed in our study is also consistent with one of the mechanisms of the “Low-Fertility Trap” hypothesis: namely, that a decline in actual family size can lead to a decline in ideal family size in the subsequent generation (Goldstein et al., 2003; Lutz et al., 2006; Testa & Grilli, 2006). Various models of fertility decision-making postulate the link between fertility ideals, intentions, and actual fertility. The “Low-Fertility Trap” hypothesis (Goldstein et al., 2003; Lutz et al., 2006; Testa & Grilli, 2006), however, directly links prolonged changes in actual fertility to potential changes in fertility ideals. This, in turn, forms part of a series of interlinked mechanisms which render increasing fertility rates ever more difficult. While the hypothesis was initially inspired by the sub-replacement fertility ideals found in German-speaking parts of Europe at the turn of the twentieth-century (Goldstein et al., 2003), there has been almost no empirical testing of this relationship between prolonged decline in actual fertility and fertility ideals. A previous study (Testa & Grilli, 2006) has shown that, in low-fertility societies in Europe, the *macrolevel* cultural context and value climate one grew up in are associated with one’s ideal family sizes. In our study, using the Chinese case, we further show that *microlevel* declines in sibship sizes and the family environment one grew up in can lead to decreases in fertility ideals. In other words, our study represents one of the very first cases of a direct, causal intergenerational link between changes in actual family size and ideal family size—a key component of the “Low Fertility Trap” hypothesis.

What explains this microlevel effect of sibship size on fertility ideals? While our data do not allow us to test mechanisms directly, drawing on the long-standing literature on intergenerational associations of family size, we argue that our estimated effect is best explained by the socialisation process experienced by only children in the families they grew up in (Axinn et al., 1994; Duncan et al., 1965; Hendershot, 1969; Johnson & Stokes, 1976). Past research has shown that only children in urban China grew up receiving high levels of parental investment of time and money on their education and health, while also facing high expectations and pressures for their social and financial success (Fong, 2004; Greenhalgh, 2008, p. 241; Short et al., 2001). The meaning and characteristics of parent–child relationships also differ for only children and those with siblings (Deutsch, 2006; Fong, 2004, 2007; Hesketh et al., 2003; Liu, 2016). As only children form their family size ideals as adults, they may have learned from the social interactions and role relationships from their early experience (Duncan et al., 1965, p. 514). From their parents’ behaviours, they may also have perceived the notion of “the priceless child”—emotionally “precious” to parents and yet economically “useless”, and even costly (Liu, 2016). This socialisation and social learning process thus helps explain how growing up with no siblings can reduce the ideal family size of only children. An alternative explanation is that only children may have attained more education as a result of receiving high levels of parental investment, and their lower fertility ideals might be a result of their higher educational attainment rather than the socialisation process. While this explanation is plausible, empirical evidence is mixed. Particularly, previous studies have shown that sibship size does not significantly impact educational attainment in urban China (Li et al., 2008) and that increased educational attainment can lead to an *increase* in ideal family size as China reaches below-replacement fertility (Chen, 2022). Future research should further explore the mechanisms underlying the sibship effect on fertility ideals and investigate other attitudes, preferences, and ideals subject to the socialisation and social learning process.

Is the sibship size effect on fertility ideals unique to China? The fuzzy RD design used in our study identifies the effect of increased probability of being an only child *due to the introduction of the OCP* (Hahn et al., 2001). Indeed, compared to other post-transitional societies, low fertility in China was driven by many more one-child families, and this model was backed up and systematically pressed through policy and propaganda (Gietel-Basten, 2019a). Despite this, China is far from the only country to have implemented strongly anti-natalist policies (Gietel-Basten, 2019a; Tsui, 2001). According to the United Nations Population Policy database, in 1976, 40 out of 156 countries had policies to lower fertility (de Silva & Tenreyro, 2017; Tsui, 2001). By 1996, 82 countries had a policy to reduce fertility, covering 70 percent of the world’s population (de Silva & Tenreyro, 2017). While China’s OCP is often considered an extreme case, fertility reduction policies in other countries have also demonstrated varying program strengths and levels of government sponsorship (Entwisle, 1989; Mauldin & Ross, 1991; Tsui, 2001). While the direct impact of anti-natalist polices on fertility and fertility preferences has been much studied and debated, in recent decades, there has been renewed attention, in China and beyond, on their long-term, intergenerational impact. Park et al. (2023) uncovered the distinct pattern of the decline to low fertility in India and drew attention to its history of coercive and forced sterilisation as a potential driver. De Silva and Tenreyro (2017) presented cross-country evidence to argue that concerted population control policies implemented in developing countries may have played a central role in the global decline in fertility rates observed today and can explain some patterns of the global fertility decline that are not well accounted for by other socioeconomic factors. Our study suggests that China’s OCP can have an intergenerational impact on personal fertility ideals, at least among individuals born right around 1980. Future research should continue to test if similar effects exist in other low-fertility societies and shed light on the specific mechanisms underlying the intergenerational effects of population control policies.

More broadly, in many parts of the world, including China, policymakers are attempting to grapple with the perceived challenges of low fertility, especially as it affects population ageing and ultimately decline. Various policy formulations are being implemented, including cash transfers and incentives (including “baby bonuses”, tax and mortgage relief); investment in childcare and childcare facilities; promoting family-friendly workplaces and so on. The ultimate goal of these policies is to raise fertility: seeking a “demographic solution to a demographic problem”. Some hope for the efficacy of these policies has been drawn from the stylised fact that in the post-transitional countries, desired fertility tends to be higher than actual fertility (Basten & Verropoulou, 2015; Bongaarts, 2001, 2002; Hagewen & Morgan, 2005; Sobotka & Beaujouan, 2014). This means that there is an “unmet demand” for children in which policies can work to realise higher fertility (Chesnais, 1996, 2000). However, the extent to which this “unmet demand” is guaranteed and not subject to change over time has not really been systematically explored. Rather, the concept of a sustained, general two-child norm is primarily based on prevailing social norms and a relatively short demographic history. Our evidence from China shows that there is no such thing as an “inevitable” or “natural” two-child norm in countries in the later stages of fertility transition—albeit under the particular conditions of the birth control restrictions of the past decades.

What does this mean for policy? Firstly, our findings should finally dispel the myth that there is some easy solution to raise fertility rates through finding some simplistic, magic formula. Rather, policies should focus more on some of the key institutional reforms which are needed to tackle the challenges of population ageing and stagnation, such as increasing productivity, *hukou* reform, pension/retirement age reform, and so on (Gietel-Basten et al., 2022). Secondly, fertility preferences are, of course, mediated by myriad other factors which change over the life course. These might include housing, income, career trajectory, relationships, caring responsibilities and so on. If it is made ever more difficult to start and grow a family because of institutional barriers and malfunctions, it is only natural to envisage a further decline in the overall period and, ultimately, cohort total fertility rates. In this context of lower fertility ideals, holistic policy suites which genuinely explore and address the challenges which young people and (prospective) parents face become ever more important if economies seek to avert transitioning to even lower fertility rates, which may, in turn, become ever more difficult, if not impossible to turn around.

## Figures and Tables

**Fig. 3:**
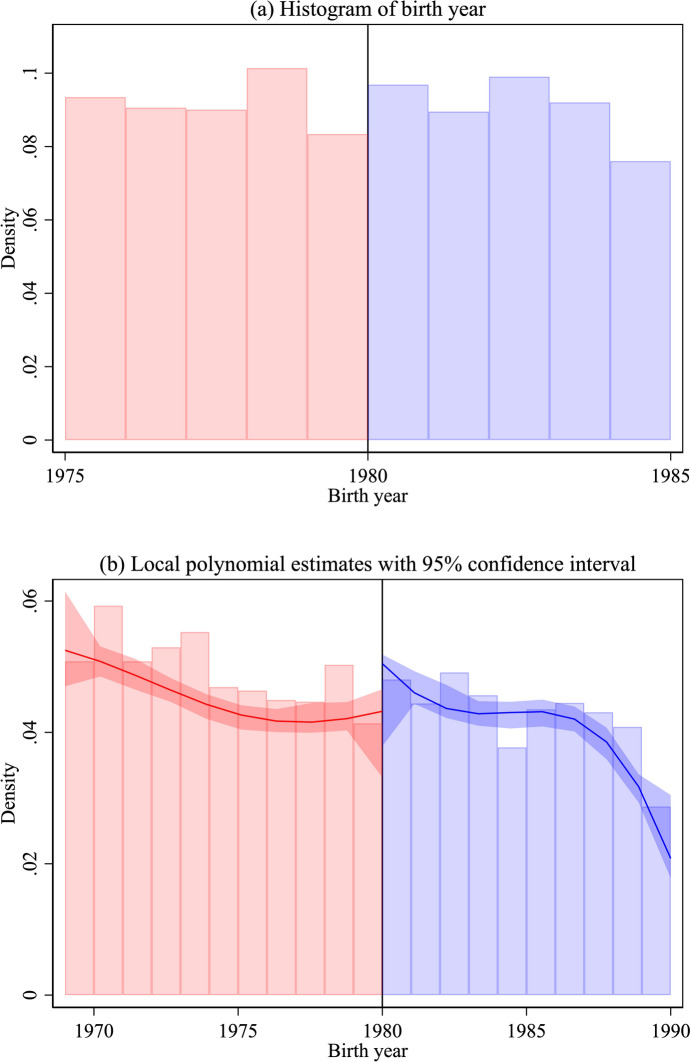
Density plots for year of birth around the cutoff

**Fig. 4:**
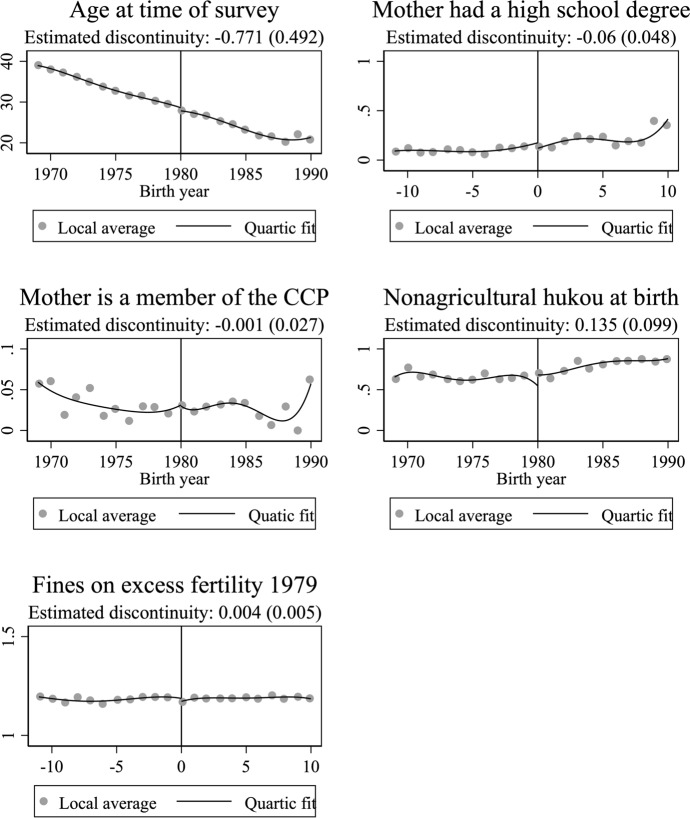
Pre-treatment variables by birth year, local average and quartic polynomial fit. Note: The dots are local averages, and the curved line plots the fitted values from a quartic regression model estimated separately on each side of the cutoff. The OCP took effect in 1980, indicated by the vertical line. Source: CGSS 2006, 2008, and 2017 (Family Module); Fine rates on excess fertility from Ebenstein (2010)

## Appendix

See Tables 5, 6, 7, 8 and Figs. 3, 4.

**Table 5:** Alternative specifications with different functional forms of birth year and windows

	(1)	(2)	(3)	(4)	(5)	(6)	(7)	(8)	(9)	(10)	(11)
First-stage: Estimated discontinuity in probabilities of being an only child											
$\widehat{{\beta }_{1}}$	0.155	0.117	0.159	0.182	0.158	0.267	0.298	0.217	0.123	0.135	0.082
t(G-1)	3.73**	3.38**	3.77**	3.62**	2.19*	4.91***	4.12**	4.61***	3.65**	3.18*	2.83*
t(G-L)	3.73**	3.38*	3.78**	3.62**	2.19†	4.92**	4.12**	4.62**	3.66*	3.18*	2.83*
Effect of being an only child on ideal family size											
$\widehat{\tau }$	− 0.641	− 0.685	− 0.610	− 0.450	− 0.804	− 0.549	− 0.631	− 0.631	− 0.702	− 0.626	− 0.690
t(G-1)	− 3.87**	− 6.93***	− 4.06**	− 2.61*	− 4.41***	− 7.13***	− 10.49***	− 12.02***	− 6.71***	− 7.67***	− 5.78**
t(G-L)	− 1.94†	− 2.25†	− 1.93†	− 1.29	− 1.90†	− 2.15†	− 2.06†	− 2.53*	− 2.12†	− 1.86	− 2.01
Effect of being an only child on desiring two or more children											
$\widehat{\tau }$	− 0.671	− 0.659	− 0.670	− 0.574	− 0.777	− 0.507	− 0.193	− 0.432	− 0.659	− 0.588	− 0.753
t(G-1)	− 3.20**	− 3.20*	− 3.28**	− 2.78*	− 1.94†	− 3.19**	− 3.61**	− 4.29**	− 3.56**	− 3.13*	− 2.88*
t(G-L)	− 3.86**	− 4.90**	− 3.71**	− 3.66**	− 3.16*	− 3.62**	− 3.73*	− 4.97**	− 3.87**	− 4.85**	− 3.75**
Birth year polynomial	Quartic	Quadratic	Quartic	Quartic	Quartic	Quartic	Quartic	Cubic	Quadratic	Quadratic	Linear
Window	[1969, 1990]	[1975, 1984]	[1969, 1991]	[1970, 1990]	[1971, 1989]	[1972, 1988]	[1973, 1987]	[1974, 1986]	[1975, 1985]	[1976, 1984]	[1977, 1983]

t(G-1) are t-statistics with degrees of freedom equal to the number of clusters minus one using individual regressions and standard errors clustered by birth year. t(G-L) are t-statistics with degrees of freedom equal to the number of clusters minus the number of group-constant variables using group mean (at birth year level) regressions and robust standard errors. All models control for birth year polynomial that is allowed to be different before and after 1980. ****p* < 0.001 ***p* < 0.01 **p* < 0.05 † < 0.10

**Table 6:** Robustness check on subsamples and controlling for period policy change

	(1)	(2)	(3)
	Below 50	Below 40	Control for end of one-child policy
	Discontinuity in being an only child		
First-stage $\widehat{{\beta }_{1}}$	0.099**	0.117***	0.097*
	(0.028)	(0.021)	(0.032)
	Ideal number of children		
Reduced form $\widehat{{\beta }_{2}}$	− 0.058*	− 0.072**	− 0.058*
	(0.025)	(0.021)	(0.024)
Fuzzy RD $\widehat{\tau }$	− 0.585***	− 0.616**	− 0.595***
	(0.107)	(0.168)	(0.091)
	Desiring two or more children		
Reduced form $\widehat{{\beta }_{2}}$	− 0.060***	− 0.071**	− 0.060***
	(0.006)	(0.016)	(0.006)
Fuzzy RD $\widehat{\tau }$	− 0.607**	− 0.606**	− 0.616*
	(0.153)	(0.149)	(0.193)
Birth year polynomial	Quadratic	Quadratic	Quadratic
Window	[1975, 1984]	[1975, 1984]	[1975, 1984]

Standard errors are in parentheses. Standard errors for first-stage and reduced form coefficients are clustered by birth year. Standard errors for fuzzy RD estimates are calculated using delta method. Model 1 replicates column 3 of Tables 2–4. All models control for birth year polynomial that is allowed to be different before and after 1980, whether mother has high school education or above, whether mother is a member of the Chinese Communist Party, whether the individual has non-agricultural hukou at birth, age of the individual at the time of the survey, fine rates on excess births in 1979 (Ebenstein, 2010), and region fixed effects. Model 3 further controls for an indicator of whether the survey year is after 2015. ****p* < 0.001 ***p* < 0.01 **p* < 0.05

**Table 7:** First-stage, reduced form, and fuzzy RD estimates by sex of the respondent

	Female	Male
	First-stage: Discontinuity in being an only child	
$\widehat{{\beta }_{1}}$	0.145**	0.145**
	(0.042)	(0.045)
	Ideal number of children	
Reduced form $\widehat{{\beta }_{2}}$	− 0.113**	− 0.089*
	(0.038)	(0.041)
Fuzzy RD $\widehat{\tau }$	− 0.776*	− 0.612*
	(0.275)	(0.227)
	Desiring two or more children	
Reduced form $\widehat{{\beta }_{2}}$	− 0.105***	− 0.104***
	(0.023)	(0.015)
Fuzzy RD $\widehat{\tau }$	− 0.726*	− 0.718**
	(0.286)	(0.240)
Birth year polynomial	Quartic	
Window	[1969, 1990]	

Standard errors are in parentheses. Standard errors for first-stage and reduced form coefficients are clustered by birth year. Standard errors for fuzzy RD estimates are calculated using delta method. All models control for birth year polynomial that is allowed to be different before and after 1980. ****p* < 0.001 ***p* < 0.01 **p* < 0.05

**Table 8:** Falsification tests: Reduced form estimates on subsamples of discontinuity in ideal number of children and probability of desiring two or more children in 1980

	(1)	(2)	(3)
	Left school before 1999	No tertiary degree	Have siblings
	Ideal number of children		
Reduced form $\widehat{{\beta }_{2}}$	− 0.343***	− 0.278***	0.046
	(0.086)	(0.062)	(0.161)
	Desiring two or more children		
Reduced form $\widehat{{\beta }_{2}}$	− 0.213***	− 0.192***	0.027
	(0.051)	(0.030)	(0.086)
Birth year polynomial	Quartic	Quartic	Quartic
Window	[1969, 1990]	[1969, 1990]	[1969, 1990]

Standard errors are in parentheses and clustered by birth year. All models control for birth year that is allowed to be different before and after 1980. ****p* < 0.001

## Footnotes

1 Ideal family size defined at the individual level is to be distinguished from fertility ideals at the societal level (Blake, 1966; Philipov & Bernardi, 2011; Ryder & Westoff, 1971; Trent, 1980), which are measured in terms of some hypothetical average individual/family and not the focus of this study. Fertility intentions are another separate concept, which has a greater sense of planning, expectation, and likely execution than ideals (either personal or societal level). Personal ideal family size is sometimes also referred to as “fertility desires” or “personal fertility ideals”.

## References

[CR1] Aassve, A., Adserà, A., Chang, P. Y., Mencarini, L., Park, H., Peng, C., Plach, S., Raymo, J. M., Wang, S., & Jean Yeung, W.-J. (2024). Family ideals in an era of low fertility. *Proceedings of the National Academy of Sciences of the United States of America,**121*(6), Article e2311847121. 10.1073/pnas.2311847121 38294942 10.1073/pnas.2311847121 PMC10861923

[CR2] Aizen, I., & Klobas, J. (2013). Fertility intentions: An approach based on the theory of planned behavior. *Demographic Research,**29*, 203–232. 10.4054/DemRes.2013.29.8

[CR3] Ajzen, I. (1991). The theory of planned behavior. *Organizational Behavior and Human Decision Processes,**50*(2), 179–211. 10.1016/0749-5978(91)90020-T

[CR4] Ajzen, I. (2012). The theory of planned behavior. In *Handbook of theories of social psychology, Vol. 1* (pp. 438–459). Sage Publications Ltd. 10.4135/9781446249215.n22

[CR5] Anastasi, A. (1956). Intelligence and family size. *Psychological Bulletin,**53*(3), 187–209. 10.1037/h0047353 13323165 10.1037/h0047353

[CR6] Anderton, D. L., Tsuya, N. O., Bean, L. L., & Mineau, G. P. (1987). Intergenerational transmission of relative fertility and life course patterns. *Demography,**24*(4), 467–480. 10.2307/2061386 3322885

[CR7] Angrist, J. D., & Krueger, A. B. (1992). The effect of age at school entry on educational attainment: An application of instrumental variables with moments from two samples. *Journal of the American Statistical Association,**87*(418), 328–336. 10.2307/2290263

[CR8] Angrist, J. D., & Pischke, J.-S. (2009). Nonstandard Standard Error Issues. In *Mostly Harmless Econometrics* (pp. 293–326). Princeton University Press; JSTOR. 10.2307/j.ctvcm4j72.15

[CR9] Angrist, J., Lavy, V., & Schlosser, A. (2010). Multiple experiments for the causal link between the quantity and quality of children. *Journal of Labor Economics,**28*(4), 773–824. 10.1086/653830

[CR10] Attané, I. (2016). Second child decisions in China. *Population and Development Review,**42*(3), 519–536. 10.1111/j.1728-4457.2016.00151.x

[CR11] Axinn, W. G., Clarkberg, M. E., & Thornton, A. (1994). Family influences on family size preferences. *Demography,**31*(1), 65–79. 10.2307/2061908 8005343

[CR12] Axinn, W. G., & Thornton, A. (1996). The influence of parents’ marital dissolutions on children’s attitudes toward family formation. *Demography,**33*(1), 66–81. 10.2307/2061714 8690141

[CR13] Banister, J. (1987). *China’s Changing Population*. Stanford University Press.

[CR14] Barber, J. S. (2000). Intergenerational influences on the entry into parenthood: Mothers’ preferences for family and nonfamily behavior. *Social Forces,**79*(1), 319–348. 10.2307/2675573

[CR15] Basten, S., & Gu, B. (2013). Childbearing preferences, reform of family planning restrictions and the Low Fertility Trap in China. *Oxford Centre for Population Research: Working Paper #61.*

[CR16] Basten, S., & Jiang, Q. (2014). China’s family planning policies: Recent reforms and future prospects. *Studies in Family Planning,**45*(4), 493–509. 10.1111/j.1728-4465.2014.00003.x 25469931 10.1111/j.1728-4465.2014.00003.x

[CR17] Basten, S., & Jiang, Q. (2015). Fertility in China: An uncertain future. *Population Studies,**69*(sup1), S97–S105. 10.1080/00324728.2014.982898 25912921 10.1080/00324728.2014.982898 PMC4440625

[CR18] Basten, S., & Verropoulou, G. (2015). A Re-Interpretation of the ‘Two-child Norm’ in Post-Transitional Demographic Systems: Fertility Intentions in Taiwan. *PLoS ONE,**10*(8), Article e0135105. 10.1371/journal.pone.0135105 26291083 10.1371/journal.pone.0135105 PMC4546404

[CR19] BBC News. (2015, October 29). China to end one-child policy and allow two. *BBC News*. https://www.bbc.com/news/world-asia-34665539

[CR20] BBC News. (2021, May 31). China allows three children in major policy shift. *BBC News*. https://www.bbc.com/news/world-asia-china-57303592

[CR21] Beaujouan, E., & Solaz, A. (2019). Is the family size of parents and children still related? Revisiting the cross-generational relationship over the last century. *Demography,**56*(2), 595–619. 10.1007/s13524-019-00767-5 30868472 10.1007/s13524-019-00767-5 PMC6449311

[CR22] Becker, G. S. (1960). An Economic Analysis of Fertility. In *Demographic and Economic Change in Developed Countries* (pp. 209–240). Columbia University Press. https://www.nber.org/books-and-chapters/demographic-and-economic-change-developed-countries/economic-analysis-fertility

[CR23] Becker, G. S., & Tomes, N. (1976). Child endowments and the quantity and quality of children. *Journal of Political Economy,**84*(4), S143–S162.

[CR24] Behrman, J. A. (2015). Does schooling affect women’s desired fertility? Evidence from Malawi, Uganda, and Ethiopia. *Demography,**52*(3), 787–809. 10.1007/s13524-015-0392-3 25951799 10.1007/s13524-015-0392-3

[CR25] Bernardi, L. (2016). The Intergenerational Transmission of Fertility. In *Emerging Trends in the Social and Behavioral Sciences* (pp. 1–16). John Wiley & Sons, Ltd. http://onlinelibrary.wiley.com/doi/abs/10.1002/9781118900772.etrds0413

[CR26] Bian, Y., & Li, L. (2012). The Chinese General Social Survey (2003–8). *Chinese Sociological Review,**45*(1), 70–97. 10.2753/CSA2162-0555450104

[CR27] Black, S. E., Devereux, P. J., & Salvanes, K. G. (2005). The more the merrier? The effect of family size and birth order on children’s education. *The Quarterly Journal of Economics,**120*(2), 669–700. 10.1093/qje/120.2.669

[CR28] Blake, J. (1966). Ideal family size among White Americans: A quarter of a century’s evidence. *Demography,**3*(1), 154–173. 10.2307/2060069

[CR29] Blake, J. (1981). Family size and the quality of children. *Demography,**18*(4), 421–442. 10.2307/2060941 7308532

[CR30] Blake, J. (1989). *Family Size and Achievement*. University of California Press.

[CR31] Bongaarts, J. (2001). Fertility and Reproductive Preferences in Post-Transitional Societies. *Population and Development Review*, *27*, 260–281. JSTOR.

[CR32] Bongaarts, J. (2002). The end of the fertility transition in the developed world. *Population and Development Review,**28*(3), 419–443.

[CR33] Brinton, M. C., Bueno, X., Oláh, L., & Hellum, M. (2018). Postindustrial fertility ideals, intentions, and gender inequality: A comparative qualitative analysis. *Population and Development Review,**44*(2), 281–309. 10.1111/padr.12128

[CR34] Cai, Y. (2010). China’s below-replacement fertility: Government policy or socioeconomic development? *Population and Development Review,**36*(3), 419–440. 10.1111/j.1728-4457.2010.00341.x 20882701 10.1111/j.1728-4457.2010.00341.x

[CR35] Cai, Y., & Feng, W. (2021). The social and sociological consequences of China’s one-child policy. *Annual Review of Sociology,**47*(Volume 47, 2021), 587–606. 10.1146/annurev-soc-090220-032839

[CR36] Calonico, S., Cattaneo, M. D., & Farrell, M. H. (2020). Optimal bandwidth choice for robust bias-corrected inference in regression discontinuity designs. *The Econometrics Journal,**23*(2), 192–210. 10.1093/ectj/utz022

[CR37] Cameron, A. C., & Miller, D. L. (2015). A practitioner’s guide to cluster-robust inference. *The Journal of Human Resources,**50*(2), 317–372. 10.3368/jhr.50.2.317

[CR38] Cameron, L., Erkal, N., Gangadharan, L., & Meng, X. (2013). Little emperors: Behavioral impacts of China’s One-Child Policy. *Science,**339*(6122), 953–957. 10.1126/science.1230221 23306438 10.1126/science.1230221

[CR39] Cattaneo, M. D., Idrobo, N., & Titiunik, R. (2019). A Practical Introduction to Regression Discontinuity Designs: Foundations. *Elements in Quantitative and Computational Methods for the Social Sciences*. 10.1017/9781108684606

[CR40] Cattaneo, M. D., Idrobo, N., & Titiunik, R. (2023). *A Practical Introduction to Regression Discontinuity Designs: Extensions* (No. arXiv:2301.08958). arXiv. 10.48550/arXiv.2301.08958

[CR41] Cattaneo, M. D., Jansson, M., & Ma, X. (2020). Simple local polynomial density estimators. *Journal of the American Statistical Association,**115*(531), 1449–1455. 10.1080/01621459.2019.1635480

[CR42] Chen, S. (2022). The positive effect of women’s education on fertility in low-fertility China. *European Journal of Population,**38*(1), 125–161. 10.1007/s10680-021-09603-2 35370527 10.1007/s10680-021-09603-2 PMC8924343

[CR43] Chen, S., & Gietel-Basten, S. (2022). How genuine are sub-replacement ideal family sizes in urban China? *Population Studies*. http://eprints.lse.ac.uk/118545/10.1080/00324728.2023.2194670 PMC10556199 37021613

[CR44] Chesnais, J.-C. (1996). Fertility, family, and social policy in contemporary Western Europe. *Population and Development Review,**22*(4), 729–739. 10.2307/2137807

[CR45] Chesnais, J.-C. (2000). *Determinants of below-replacement fertility* (No. Special Issue Nox. 40/41; Population Bulletin of the United Nations, p. 6). United Nations Population Division.

[CR46] Conley, D., & Glauber, R. (2006). Parental educational investment and children’s academic risk: Estimates of the impact of sibship size and birth order from exogenous variation in fertility. *Journal of Human Resources,**XL I*(4), 722–737. 10.3368/jhr.XLI.4.722

[CR47] Cools, S., & Kaldager Hart, R. (2017). The effect of childhood family size on fertility in adulthood: New evidence from IV estimation. *Demography,**54*(1), 23–44. 10.1007/s13524-016-0537-z 28032264 10.1007/s13524-016-0537-z

[CR48] Dahlberg, J. (2013). Family influence in fertility: A longitudinal analysis of sibling correlations in first birth risk and completed fertility among Swedish men and women. *Demographic Research,**29*(9), 233–246. 10.4054/DemRes.2013.29.9

[CR49] de Silva, T., & Tenreyro, S. (2017). Population control policies and fertility convergence. *Journal of Economic Perspectives,**31*(4), 205–228. 10.1257/jep.31.4.205 10.1257/jep.31.4.205 29465218

[CR50] Deutsch, F. M. (2006). Filial piety, patrilineality, and China’s one-child policy. *Journal of Family Issues,**27*(3), 366–389. 10.1177/0192513X05283097

[CR51] Donald, S. G., & Lang, K. (2007). Inference with difference-in-differences and other panel data. *The Review of Economics and Statistics,**89*(2), 221–233.

[CR52] Duncan, O. D., Freedman, R., Coble, J. M., & Slesinger, D. P. (1965). Marital fertility and size of family of orientation. *Demography,**2*(1), 508–515. 10.2307/2060135

[CR53] Ebenstein, A. (2010). The “Missing Girls” of China and the Unintended Consequences of the One Child Policy. *The Journal of Human Resources,**45*(1), 87–115. 10.3368/jhr.45.1.87

[CR54] Entwisle, B. (1989). Measuring components of family planning program effort. *Demography,**26*(1), 53–76. 10.2307/2061493 2737358

[CR55] Fasang, A. E., & Raab, M. (2014). Beyond transmission: Intergenerational patterns of family formation among middle-class American families. *Demography,**51*(5), 1703–1728. 10.1007/s13524-014-0322-9 25145325 10.1007/s13524-014-0322-9

[CR56] Feng, W., Cai, Y., & Gu, B. (2013). Population, policy, and politics: How will history judge China’s one-child policy? *Population and Development Review,**38*(s1), 115–129. 10.1111/j.1728-4457.2013.00555.x

[CR57] Fong, V. L. (2004). *Only Hope*. Stanford University Press. https://www.sup.org/books/asian-studies/only-hope

[CR58] Fong, V. L. (2007). Parent-child communication problems and the perceived inadequacies of Chinese only children. *Ethos,**35*(1), 85–127. 10.1525/eth.2007.35.1.85

[CR59] Fort, M., Schneeweis, N., & Winter-Ebmer, R. (2016). Is education always reducing fertility? Evidence from compulsory schooling reforms. *The Economic Journal,**126*(595), 1823–1855. 10.1111/ecoj.12394

[CR60] Gao, S., & Wang, T. (2025). A systematic review of research on women’s education and fertility in China: Implications for addressing demographic changes. *Asian Population Studies*, *0*(0), 1–18. 10.1080/17441730.2025.2538918

[CR61] Gelman, A., & Imbens, G. (2019). Why high-order polynomials should not be used in regression discontinuity designs. *Journal of Business & Economic Statistics,**37*(3), 447–456. 10.1080/07350015.2017.1366909

[CR62] Gietel-Basten, S. (2019a). Chapter 4. Fertility Preferences in Low-Fertility Pacific Asia. In *The “Population Problem” in Pacific Asia*. Oxford University Press.

[CR63] Gietel-Basten, S. (2019b). *The ‘Population Problem’ in Pacific Asia*. Oxford University Press.

[CR64] Gietel-Basten, S., Rotkirch, A., & Sobotka, T. (2022). Changing the perspective on low birth rates: Why simplistic solutions won’t work. *BMJ (Clinical Research Ed.),**379*, Article e072670. 10.1136/bmj-2022-072670 10.1136/bmj-2022-072670 PMC10915774 36379520

[CR65] Girard, A., & Roussel, L. (1982). Ideal family size, fertility, and population policy in Western Europe. *Population and Development Review,**8*(2), 323–345. 10.2307/1972989

[CR66] Glass, J., Bengtson, V. L., & Dunham, C. C. (1986). Attitude similarity in three-generation families: Socialization, status inheritance, or reciprocal influence? *American Sociological Review,**51*(5), 685–698. 10.2307/2095493

[CR67] Global Times. (2022, August 16). *17 Chinese govt departments issue guideline to boost population growth amid falling birth rate—Global Times*. https://www.globaltimes.cn/page/202208/1273160.shtml

[CR68] Goldstein, J., Lutz, W., & Testa, M. R. (2003). The emergence of sub-replacement family size ideals in Europe. *Population Research and Policy Review,**22*(5), 479–496. 10.1023/B:POPU.0000020962.80895.4a

[CR69] Goodkind, D. (2017). The astonishing population averted by China’s birth restrictions: Estimates, nightmares, and reprogrammed ambitions. *Demography,**54*(4), 1375–1400. 10.1007/s13524-017-0595-x 28762036 10.1007/s13524-017-0595-x

[CR70] Greenhalgh, S. (1986). Shifts in China’s population policy, 1984–86: Views from the Central, Provincial, and Local Levels. *Population and Development Review,**12*(3), 491–515. 10.2307/1973220

[CR71] Greenhalgh, S. (2008). *Just one child: Science and policy in Deng’s China*. University of California Press.

[CR72] Greenhalgh, S. (2018). Making demography astonishing: Lessons in the politics of population science. *Demography,**55*(2), 721–731. 10.1007/s13524-018-0660-0 29623607 10.1007/s13524-018-0660-0

[CR73] Gu, B., Wang, F., Guo, Z., & Erli, Z. (2007). China’s local and national fertility policies at the end of the twentieth century. *Population and Development Review,**33*(1), 129–148. 10.1111/j.1728-4457.2007.00161.x

[CR74] Guo, Z., Gietel-Basten, S., & Gu, B. (2019). The lowest fertility rates in the world? Evidence from the 2015 Chinese 1% sample census. *China Population and Development Studies,**2*(3), 245–258. 10.1007/s42379-018-0012-1

[CR75] Hagewen, K. J., & Morgan, S. P. (2005). Intended and ideal family size in the United States, 1970–2002. *Population and Development Review,**31*(3), 507–527. 10.1111/j.1728-4457.2005.00081.x 20376334 10.1111/j.1728-4457.2005.00081.x PMC2849141

[CR76] Hahn, J., Todd, P., & Van der Klaauw, W. (2001). Identification and estimation of treatment effects with a regression-discontinuity design. *Econometrica,**69*(1), 201–209.

[CR77] Hazan, M., & Zoabi, H. (2015). Do highly educated women choose smaller families? *The Economic Journal,**125*(587), 1191–1226. 10.1111/ecoj.12148

[CR78] Heiland, F., Prskawetz, A., & Sanderson, W. C. (2008). Are individuals’ desired family sizes stable? Evidence from West German panel data. *European Journal of Population / Revue Européenne De Démographie,**24*(2), 129–156. 10.1007/s10680-008-9162-x

[CR79] Hendershot, G. E. (1969). Familial satisfaction, birth order, and fertility values. *Journal of Marriage and Family,**31*(1), 27–33. 10.2307/350003

[CR80] Hesketh, T., Qu, J., & Tomkins, A. (2003). Health effects of family size: Cross sectional survey in Chinese adolescents. *Archives of Disease in Childhood,**88*(6), 467–471. 10.1136/adc.88.6.467 12765907 10.1136/adc.88.6.467 PMC1763126

[CR81] Hoem, B., & Hoem, J. M. (1989). The impact of women’s employment on second and third births in modern Sweden. *Population Studies,**43*(1), 47–67. 10.1080/0032472031000143846

[CR82] Hou, J. (2015). Changes in the Chinese population’s fertility intentions: 1980–2011. *Social Sciences in China,**36*(1), 46–63. 10.1080/02529203.2015.1001482

[CR83] Huang, W., Lei, X., & Zhao, Y. (2015). One-child policy and the rise of man-made twins. *The Review of Economics and Statistics,**98*(3), 467–476. 10.1162/REST_a_00567

[CR84] Imbens, G., & Lemieux, T. (2008). Regression discontinuity designs: A guide to practice. *Journal of Econometrics,**142*, 615–635.

[CR85] Inoue, A., & Solon, G. (2010). Two-sample instrumental variables estimators. *The Review of Economics and Statistics,**92*(3), 557–561.

[CR86] Jennings, E. A., Axinn, W. G., & Ghimire, D. J. (2012). The effect of parents’ attitudes on sons’ marriage timing. *American Sociological Review,**77*(6), 923–945. 10.1177/0003122412464041 23483623 10.1177/0003122412464041 PMC3590910

[CR87] Jiang, Q., Li, Y., & Sánchez-Barricarte, J. J. (2016). Fertility intention, son preference, and second childbirth: Survey findings from Shaanxi Province of China. *Social Indicators Research,**125*(3), 935–953. 10.1007/s11205-015-0875-z 28769144 10.1007/s11205-015-0875-z PMC5536174

[CR88] Johnson, N. E., & Stokes, C. S. (1976). Family size in successive generations: The effects of birth order, intergenerational change in lifestyle, and familial satisfaction. *Demography,**13*(2), 175–187. 10.2307/2060799 1278578

[CR89] Kohler, H.-P., Rodgers, J. L., & Christensen, K. (1999). Is fertility behavior in our genes? Findings from a Danish twin study. *Population and Development Review,**25*(2), 253–288. 10.1111/j.1728-4457.1999.00253.x

[CR90] Kolk, M. (2015). The causal effect of an additional sibling on completed fertility: An estimation of intergenerational fertility correlations by looking at siblings of twins. *Demographic Research,**32*, 1409–1420.

[CR91] Kravdal, Ø. (1992). The emergence of a positive relation between education and third birth rates in Norway with supportive evidence from the United States. *Population Studies,**46*(3), 459–475. 10.1080/0032472031000146456

[CR92] Kravdal, Ø., & Rindfuss, R. R. (2008). Changing Relationships between Education and Fertility: A Study of Women and Men Born 1940 to 1964. *American Sociological Review*, *73*(5), 854–873. JSTOR.

[CR93] Kugler, A. D., & Kumar, S. (2017). Preference for boys, family size, and educational attainment in India. *Demography,**54*(3), 835–859. 10.1007/s13524-017-0575-1 28484996 10.1007/s13524-017-0575-1 PMC5486858

[CR94] Lee, D. S. (2008). Randomized experiments from non-random selection in U.S. house elections. *Journal of Econometrics,**142*(2), 675–697. 10.1016/j.jeconom.2007.05.004

[CR95] Lee, D. S., & Lemieux, T. (2010). Regression discontinuity designs in economics. *Journal of Economic Literature,**48*(2), 281–355. 10.1257/jel.48.2.281

[CR96] Li, H., Xue, M., Hellerstein, S., Cai, Y., Gao, Y., Zhang, Y., Qiao, J., Blustein, J., & Liu, J. (2019). Association of China’s universal two child policy with changes in births and birth related health factors: National, descriptive comparative study. *BMJ*, *366*. 10.1136/bmj.l4680 10.1136/bmj.l4680 PMC6699592 31434652

[CR97] Li, H., Yi, J., & Zhang, J. (2011). Estimating the effect of the One-Child Policy on the sex ratio imbalance in China: Identification based on the difference-in-differences. *Demography,**48*(4), 1535–1557. 21853400 10.1007/s13524-011-0055-y

[CR98] Li, H., Zhang, J., & Zhu, Y. (2008). The quantity-quality trade-off of children in a developing country: Identification using Chinese twins. *Demography,**45*(1), 223–243. 10.1353/dem.2008.0006 18390301 10.1353/dem.2008.0006 PMC2831373

[CR99] Liang, Z. (2014). 艰难的历程:从“一胎化”到“女儿户”. *开放时代 [Open Times]*, *3*.

[CR100] Liu, F. (2016). The Rise of the “Priceless” Child in China. *Comparative Education Review,**60*(1), 105–130. 10.1086/684457

[CR101] Liu, X. (2025). Fertility intentions of China’s one-child generation: A comparative analysis with the US. *Journal of Family Studies,**31*(1), 1–34. 10.1080/13229400.2024.2399263

[CR102] Lutz, W., Skirbekk, V., & Testa, M. R. (2006). The low-fertility trap hypothesis: Forces that may lead to further postponement and fewer births in Europe. *Vienna Yearbook of Population Research,**4*, 167–192.

[CR103] Mauldin, W. P., & Ross, J. A. (1991). Family planning programs: Efforts and results, 1982–89. *Studies in Family Planning,**22*(6), 350–367. 10.2307/1966449 1792675

[CR104] McCrary, J. (2008). Manipulation of the running variable in the regression discontinuity design: A density test. *Journal of Econometrics,**142*(2), 698–714. 10.1016/j.jeconom.2007.05.005

[CR105] Merli, M. G., & Morgan, S. P. (2011). Below replacement fertility preferences in Shanghai. *Population (Paris),**66*(3), 519–542. 10.3917/pope.1103.0519 PMC3769784 24039621

[CR106] Merli, M. G., & Smith, H. I. (2002). Has the Chinese family planning policy been successful in changing fertility preferences? *Demography,**39*(3), 557–572. 10.1353/dem.2002.0029 12205758 10.1353/dem.2002.0029

[CR107] Mills, M. C., & Tropf, F. C. (2015). The biodemography of fertility: A review and future research frontiers. *Kölner Zeitschrift Für Soziologie und Sozialpsychologie,**67*(Suppl 1), 397–424. 10.1007/s11577-015-0319-4 26412877 10.1007/s11577-015-0319-4 PMC4577548

[CR108] Morgan, S. P., Zhigang, G., & Hayford, S. R. (2009). China’s below-replacement fertility: Recent trends and future prospects. *Population and Development Review,**35*(3), 605–629. 10.1111/j.1728-4457.2009.00298.x 20376285 10.1111/j.1728-4457.2009.00298.x PMC2849170

[CR109] Morosow, K., & Kolk, M. (2020). How Does Birth Order and Number of Siblings Affect Fertility? A Within-Family Comparison Using Swedish Register Data. *European Journal of Population,**36*(2), 197–233. 10.1007/s10680-019-09525-0 32256257 10.1007/s10680-019-09525-0 PMC7113329

[CR110] Müller, M. W., Hamory, J., Johnson-Hanks, J., & Miguel, E. (2022). The illusion of stable fertility preferences. *Population Studies,**76*(2), 169–189. 10.1080/00324728.2022.2057577 35576966 10.1080/00324728.2022.2057577 PMC9256780

[CR111] Murphy, M. (1999). Is the relationship between fertility of parents and children really weak? *Social Biology,**46*(1–2), 122–145. 10.1080/19485565.1999.9988991 10842505 10.1080/19485565.1999.9988991

[CR112] Murphy, M. (2013). The intergenerational transmission of reproductive behaviour: Comparative perspectives. *The History of the Family,**18*(2), 107–115. 10.1080/1081602X.2013.808447

[CR113] Murphy, M., & Knudsen, L. B. (2002). The intergenerational transmission of fertility in contemporary Denmark: The effects of number of siblings (full and half), birth order, and whether male or female. *Population Studies,**56*(3), 235–248. 10.1080/00324720215937 12553320 10.1080/00324720215937

[CR114] Murphy, M., & Wang, D. (2001). Family-level continuities in childbearing in low-fertility societies. *European Journal of Population / Revue Européenne De Démographie,**17*(1), 75–96. 10.1023/A:1010744314362

[CR115] National Bureau of Statistics of China. (2023, January 17). *National Economy Withstood Pressure and Reached a New Level in 2022*. http://www.stats.gov.cn/english/PressRelease/202301/t20230117_1892094.html

[CR116] Nie, Y., & Wyman, R. J. (2005). The One-Child Policy in Shanghai: Acceptance and Internalization. *Population and Development Review*, *31*(2), 313–336. JSTOR.

[CR117] Nisén, J., Klüsener, S., Dahlberg, J., Dommermuth, L., Jasilioniene, A., Kreyenfeld, M., Lappegård, T., Li, P., Martikainen, P., Neels, K., Riederer, B., te Riele, S., Szabó, L., Trimarchi, A., Viciana, F., Wilson, B., & Myrskylä, M. (2021). Educational differences in cohort fertility across sub-national regions in Europe. *European Journal of Population,**37*(1), 263–295. 10.1007/s10680-020-09562-0 33597840 10.1007/s10680-020-09562-0 PMC7864854

[CR118] Nisén, J., Martikainen, P., Kaprio, J., & Silventoinen, K. (2013). Educational differences in completed fertility: A behavioral genetic study of Finnish male and female twins. *Demography,**50*(4), 1399–1420. 10.1007/s13524-012-0186-9 23344794 10.1007/s13524-012-0186-9

[CR119] Park, N., Vyas, S., Broussard, K., & Spears, D. (2023). Near-universal marriage, early childbearing, and low fertility: India’s alternative fertility transition. *Demographic Research,**48*, 945–956. 10.4054/DemRes.2023.48.34 38288421 10.4054/DemRes.2023.48.34 PMC10824390

[CR120] People’s Congress. (2021, March). *Outline of the People’s Republic of China 14th Five-Year Plan for National Economic and Social Development and Long-Range Objectives for 2035*. Xinhua News Agency. http://www.gov.cn/xinwen/2021-03/13/content_5592681.htm

[CR121] Philipov, D., & Bernardi, L. (2011). Concepts and Operationalisation of Reproductive Decisions Implementation in Austria, Germany and Switzerland. *Comparative Population Studies*, *36*(2–3). 10.12765/CPoS-2011-14

[CR122] Ponczek, V., & Souza, A. P. (2012). New evidence of the causal effect of family size on child quality in a developing country. *Journal of Human Resources,**47*(1), 64–106. 10.3368/jhr.47.1.64

[CR123] Qian, N. (2009). *Quantity-Quality and the One Child Policy:The Only-Child Disadvantage in School Enrollment in Rural China* (No. w14973). National Bureau of Economic Research. 10.3386/w14973

[CR124] Ray, C., Harcey, S., Greil, A., Tiemeyer, S., & McQuillan, J. (2018). Stability and change in personal fertility ideals among U.S. women in heterosexual relationships. *Demographic Research,**39*, 459–486. 10.4054/DemRes.2018.39.16 10.4054/demres.2018.39.16 PMC13186419 42163906

[CR125] Régnier-Loilier, A. (2006). Influence of Own Sibship Size on the Number of Children Desired at Various Times of Life: The Case of France. *Population (English Edition, 2002-)*, *61*(3), 165–194.

[CR126] Rosenzweig, M. R., & Wolpin, K. I. (1980). Testing the quantity-quality fertility model: The use of twins as a natural experiment. *Econometrica,**48*(1), 227–240. 10.2307/1912026 12261749

[CR127] Rosenzweig, M. R., & Zhang, J. (2009). Do Population Control Policies Induce More Human Capital Investment? Twins, Birth Weight and China’s “One-Child” Policy. *The Review of Economic Studies,**76*(3), 1149–1174. 10.1111/j.1467-937X.2009.00563.x

[CR128] Ryder, N. B., & Westoff, C. F. (1971). Orientations Toward Numbers of Children. In *Reproduction in the U.S., 1965* (pp. 19–36). Princeton University Press; JSTOR. 10.2307/j.ctt1m3nws9.4

[CR129] Savelieva, K., Nitsche, N., Berg, V., Miettinen, A., Rotkirch, A., & Jokela, M. (2021). *Birth cohort changes in fertility ideals: Evidence from repeated cross-sectional surveys in Finland*. SocArXiv. 10.31235/osf.io/7vtqm

[CR130] Short, S. E., Fengying, Z., Siyuan, X., & Mingliang, Y. (2001). China’s one-child policy and the care of children: An analysis of qualitative and quantitative data*. *Social Forces,**79*(3), 913–943. 10.1353/sof.2001.0025

[CR131] Sobotka, T., & Beaujouan, É. (2014). Two is best? The persistence of a two-child family ideal in Europe. *Population and Development Review,**40*(3), 391–419. 10.1111/j.1728-4457.2014.00691.x

[CR132] Testa, M. R. (2014). On the positive correlation between education and fertility intentions in Europe: Individual- and country-level evidence. *Advances in Life Course Research,**21*, 28–42. 10.1016/j.alcr.2014.01.005 26047540 10.1016/j.alcr.2014.01.005 PMC4477715

[CR133] Testa, M. R., & Grilli, L. (2006). The influence of childbearing regional contexts on ideal family size in Europe. *Population (English Edition, 2002-),**61*(1/2), 109–137.

[CR134] Thornton, A. (1980). The influence of first generation fertility and economic status on second generation fertility. *Population and Environment,**3*(1), 51–72. 10.1007/BF01253070

[CR135] Trent, R. B. (1980). Evidence bearing on the construct validity of ‘ideal family size.’ *Population and Environment,**3*(3), 309–327. 10.1007/BF01255345

[CR136] Trinitapoli, J., & Yeatman, S. (2018). The flexibility of fertility preferences in a context of uncertainty. *Population and Development Review,**44*(1), 87–116. 10.1111/padr.12114 29695890 10.1111/padr.12114 PMC5900734

[CR137] Tsui, A. O. (2001). Population policies, family planning programs, and fertility: The record. *Population and Development Review,**27*, 184–204.

[CR138] UNFPA. (2024). *State of World Population report 2024*. United Nations Population Fund. https://www.unfpa.org/swp2024

[CR139] United Nations Department of Economic and Social Affairs, Population Division. (2024). *World Population Prospects: The 2024 Revision*. https://population.un.org/wpp/

[CR140] Wang, F., Cai, Y., Shen, K., & Gietel-Basten, S. (2018). Is demography just a numerical exercise? Numbers, politics, and legacies of China’s one-child policy. *Demography,**55*(2), 693–719. 10.1007/s13524-018-0658-7 29623606 10.1007/s13524-018-0658-7

[CR141] Wang, X. (2023, March 10). Experts propose ideas for reversing declining births. *China Daily*. https://www.chinadaily.com.cn/a/202303/10/WS640a811da31057c47ebb3734.html

[CR142] Whyte, M. K., & Gu, S. Z. (1987). Popular response to China’s fertility transition. *Population and Development Review,**13*(3), 471–493. 10.2307/1973135

[CR143] Wood, J., Klüsener, S., Neels, K., & Myrskylä, M. (2020). Shifting links in the relationship between education and fertility. *Population, Space and Place,**26*(8), Article e2342. 10.1002/psp.2342

[CR144] Wood, J., Neels, K., & Kil, T. (2014). The educational gradient of childlessness and cohort parity progression in 14 low fertility countries. *Demographic Research*, *31*, 1365–1416. JSTOR.

[CR145] Yeatman, S., Sennott, C., & Culpepper, S. (2013). Young women’s dynamic family size preferences in the context of transitioning fertility. *Demography,**50*(5), 1715–1737. 10.1007/s13524-013-0214-4 23619999 10.1007/s13524-013-0214-4 PMC3786023

[CR146] Yeung, J. (2021). *These Chinese villages are paying couples to have more children*. CNN. https://www.cnn.com/2021/09/24/china/three-child-cash-incentive-intl-hnk/index.html

[CR147] Zeng, Y., & Hesketh, T. (2016). The effects of China’s universal two-child policy. *The Lancet,**388*(10054), 1930–1938. 10.1016/S0140-6736(16)31405-2 10.1016/S0140-6736(16)31405-2 PMC5944611 27751400

[CR148] Zhang, H. (2007). From Resisting to ‘Embracing?’ the One-Child Rule: Understanding New Fertility Trends in a Central China Village. *The China Quarterly,**192*, 855–875.

[CR149] Zhang, J. (2017). The evolution of China’s one-child policy and its effects on family outcomes. *Journal of Economic Perspectives,**31*(1), 141–160. 10.1257/jep.31.1.141

[CR150] Zhao, Z., & Zhang, G. (2018). Socioeconomic factors have been the major driving force of China’s fertility changes since the mid-1990s. *Demography,**55*(2), 733–742. 10.1007/s13524-018-0662-y 29623608 10.1007/s13524-018-0662-y

[CR151] Zheng, Z., Gu, B., & Gietel-Basten, S. (2018). Fertility Preferences in China. In S. Gietel-Basten, J. Casterline, & M. K. Choe (Eds), *Family Demography in Asia*. Edward Elgar Publishing.

[CR152] Zhenzhen, Z., Cai, Y., Feng, W., & Baochang, G. (2009). Below-replacement fertility and childbearing intention in Jiangsu province. *CHINA. Asian Population Studies,**5*(3), 329–347. 10.1080/17441730903351701

[CR153] Zhuang, Y., Jiang, Y., & Li, B. (2020). Fertility intention and related factors in China: Findings from the 2017 National Fertility Survey. *China Population and Development Studies,**4*(1), 114–126. 10.1007/s42379-020-00053-7

